# Material‐Efficient Synthesis of Polymer Membrane‐Immobilized Metal Oxides for Advanced Oxidation Processes: Catalytic Mechanism, Stability and Performance

**DOI:** 10.1002/smll.202507532

**Published:** 2025-08-15

**Authors:** S. Amir H. Hesaraki, Burcu Önal, Oleg Prymak, Zhijun Ren, Mathias Ulbricht, Lukas Fischer

**Affiliations:** ^1^ Lehrstuhl für Technische Chemie II and Center for Water and Environmental Research (ZWU) Universität Duisburg‐Essen 45141 Essen Germany; ^2^ Center for Nanointegration Duisburg‐Essen (CENIDE) University Duisburg‐Essen 47057 Duisburg Germany; ^3^ Inorganic Chemistry University Duisburg‐Essen 45141 Essen Germany; ^4^ Tianjin Key Laboratory of Clean Energy and Pollutant Control Hebei University of Technology Tianjin 300401 China

**Keywords:** catalyst modeling, heterogeneous catalysis, metal immobilization, pollutant degradation, water treatment

## Abstract

A unique *all‐in‐one* synthesis is presented for membrane‐immobilized transition metal oxides, integrating into a single process: metallic nanoparticle synthesis within a polymer dope solution, porous support membrane formation via film casting and polymer precipitation, and aqueous room‐temperature oxidation using atmospheric oxygen. This approach achieves near‐perfect metal utilization and enables synthesis of different metal oxides under identical conditions. As‐prepared CrO_2_, MnO_2_, FeOOH, CoOOH, Ni(OH)_2_, CuO, and ZnO are benchmarked in advanced oxidation processes (AOPs) for water treatment at neutral pH and with NaCl and NaHCO_3_. Tetracycline, diclofenac, cefalexin, and *p*‐nitrophenol are tested as organic pollutants and persulfate (S_2_O_8_^2−^), hydrogen peroxide (H_2_O_2_), and sulfite (SO_3_^2−^) as oxidants. Key reactivity trends and performance indicators across diverse catalyst‐oxidant‐pollutant systems are identified. The role of catalyst‐oxidant affinities is elucidated, showing how Lewis acid–base interactions at the metal oxide surface impact metal cation leaching and resistance to interference from dissolved anions during oxidant activation. Furthermore, a novel theoretical framework is introduced that links the material properties of metal oxides to their catalytic oxidant activation mechanisms. Building on this framework, a mathematical model is established that predicts the catalytic activity of metal oxides in AOPs across various conditions, providing a new strategy for rational catalyst design.

## Graphical abstract

This study presents a material‐efficient *all‐in‐one* platform that integrates metal nanoparticle synthesis in a polymer dope solution with subsequent membrane preparation and room‐temperature oxidation. Key transition metal oxides are benchmarked in various advanced oxidation processes (AOPs) to uncover fundamental principles of their catalytic performance. Based on these insights, a novel theoretical framework is introduced for designing catalysts for water treatment.

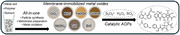

## 1 Introduction

The rapid and global rise of environmental pollution originating from human activities is one of the most critical challenges of today. The release of diverse persistent organic pollutants (POPs) into water, including pharmaceuticals and industrial residues, degrades aquatic ecosystems and promotes the emergence of antibiotic‐resistant bacteria.^[^ ^1^, ^2^, ^3^ ^]^ A major source of pollution is industrial wastewater, which can contain POP concentrations ranging from hundreds of µg L^−1^ to several mg L^−1^.^[^ ^4^, ^5^ ^]^ While treating pollution at localized industrial sources is theoretically straightforward, traditional wastewater treatment technologies are ill‐equipped to remove small, organic molecules.^[^ ^6^, ^7^ ^]^ This gap in treatment capability emphasizes the urgent need for the development of innovative approaches for water purification. One promising technology is the remediation of POPs by advanced oxidation processes (AOPs), which are based on the generation of active radical species that can degrade a wide range of organic molecules.^[^ ^8^ ^]^ As radical source, commonly employed oxidants include hydrogen peroxide (H_2_O_2_), persulfate (S_2_O_8_^2−^) and, more recently, sulfite (SO_3_^2−^), which has both oxidation and reduction capabilities.^[^ ^9^, ^10^ ^]^ However, the practical adoption of AOPs in water treatment is currently still lacking due to long treatment times and high costs.

Heterogeneous metal catalysts can substantially enhance water treatment capacities by activating oxidants, yet their advancement is hindered by fundamental challenges.^[^ ^11^, ^12^ ^]^ Current research often prioritizes development of novel, complex catalysts over material efficiency and scalability.^[^ ^13^, ^14^ ^]^ Moreover, a clear understanding of general activity trends and structure‐property relationships in catalytic oxidant activation, even for simple transition metal oxides, is lacking. This is partly because comparisons between metal oxides often rely on materials obtained by disparate synthesis methods (e.g., hydrothermal, sol–gel, precipitation) or conditions (e.g., pH, stabilizers, calcination temperature).^[^ ^15^, ^16^ ^]^ Consequently, synthesis‐related variations of physicochemical properties complicate accurate performance quantification.^[^ ^17^, ^18^ ^]^ Among these variations, agglomeration level is arguably the most impactful since metal catalysts are typically synthesized and isolated as nanoparticle powders. Not only does the synthesis process affect particle agglomeration, but different metal oxides also possess inherent variations in their proneness to agglomeration and sintering, as well as their effectiveness for redispersion in water.^[^ ^19^, ^20^ ^]^ This makes it extremely difficult to relate the observed catalytic conversion to the intrinsic specific activity of the metal oxide during AOPs.

A promising approach to overcome these challenges is to immobilize metal nanoparticles onto micro‐scale support materials, for example using porous polymer membranes as substrate.^[^ ^21^, ^22^ ^]^ Typically, porous polymer membranes are prepared through liquid non‐solvent induced phase separation (NIPS), where the polymer is dissolved in a water‐miscible solvent (“casting solution”), cast into a flat sheet or extruded as fibers, and then immersed in a water bath (“film casting *cum* phase separation”).^[^ ^23^ ^]^ This causes rapid polymer precipitation into a porous, cohesive matrix structure. There are two common approaches for incorporating metal catalysts into such membranes.^[^ ^24^, ^25^, ^26^, ^27^ ^]^ The first one involves pre‐synthesizing a particle powder, which is then blended with the polymer casting solution.^[^ ^28^, ^29^ ^]^ During the following polymer precipitation, the particles are physically incorporated into the membrane matrix, but using pre‐synthesized catalyst powders often results in their incorporation into the final membrane in an agglomerated state. The second approach involves first preparing the porous polymer membrane and then either depositing pre‐synthesized metal particles onto its (pore) surface or synthesizing the particles in the presence of the membrane.^[^ ^30^, ^31^, ^32^, ^33^, ^34^ ^]^ This approach suffers from extremely low material efficiency. Typically, only a small fraction of solid metal actually deposits onto the membrane, and these particles readily detach due to binding solely via adsorption.

Recently, we invented another method, which integrates the in situ synthesis of metallic nanoparticles from metal ions in a polymer dope solution with their rapid immobilization into porous membranes via film casting and polymer precipitation in a water bath.^[^ ^35^, ^36^ ^]^ This approach leads to strong particle attachment within the polymer matrix and mitigates particle agglomeration. However, the application of this synthesis has, to date, been limited to exemplary demonstrations with Ni, Cu, and Pd‐In decorated membranes.^[^ ^35^, ^36^, ^37^ ^]^

Therefore, our goal was to develop the *all‐in‐one* method into a material‐efficient platform for synthesizing diverse porous membrane‐immobilized transition metal oxides. To this end, we extended the integrated process of metallic nanoparticle synthesis (through reduction in the polymer casting solution) and membrane immobilization by a subsequent oxidation step. Demonstrating the versatility of this platform, we fabricated oxides of Cr, Mn, Fe, Co, Ni, Cu, and Zn. The second objective was to elucidate performance trends in AOPs for these key metal oxides. The *all‐in‐one* method uniquely enables this by ensuring that all these oxides are prepared under identical conditions and immobilized in a dispersed state within a porous matrix, thereby minimizing differences in particle agglomeration and dispersion. We benchmarked their catalytic activity using tetracycline, cefalexin, diclofenac, and *p*‐nitrophenol as organic pollutants, and persulfate, H_2_O_2_ and sulfite as oxidants. Finally, we conducted an in‐depth analysis of the factors governing performance over diverse catalyst‐pollutant‐oxidant systems, and established a novel theoretical framework for explaining and predicting the catalytic activity of transition metal oxides in AOPs.

## 2 Results and Discussion

### 2.1 *All‐In‐One Fabrication*: Solid Nanoparticles, Porous Membrane Matrix, and Metal Oxidation

**Figure 1:**
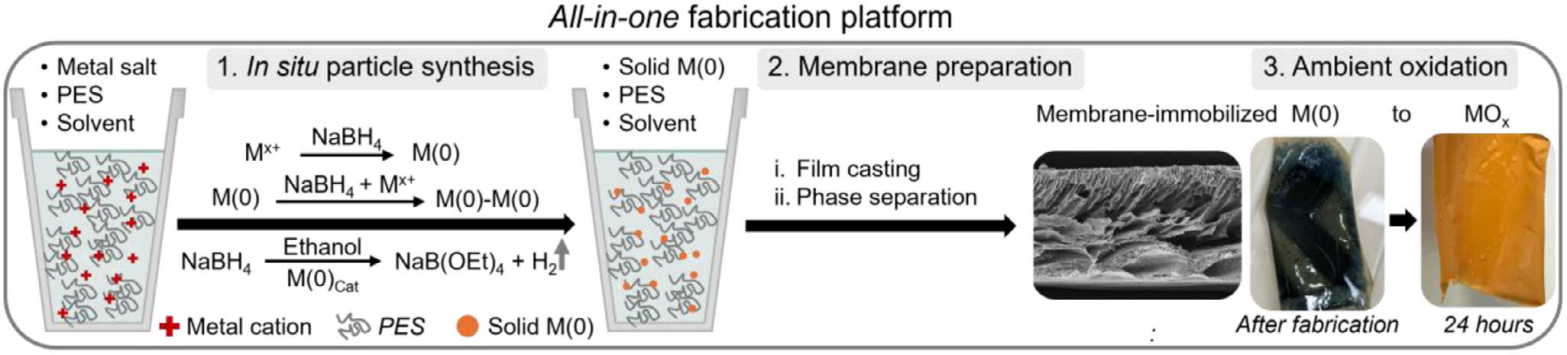
Schematic depiction of the *all‐in‐one* fabrication platform for membrane‐immobilized transition metal oxides. The exemplary images shown are those of *M_Fe*.

In this study, we extended our *all‐in‐one* method toward a platform for the synthesis of porous membrane‐immobilized oxides of Cr, Mn, Fe, Co, Ni, Cu, and Zn (**Figure** **1**).

First, solid zerovalent metal nanoparticles were synthesized via the sonochemical reduction of a dissolved metal salt using 5 mol equivalent NaBH_4_ (see in Section S1.2, Supporting Information for experimental details). This step was carried out within the casting‐reaction solution containing polyethersulfone (PES) as matrix polymer (15 wt.%) in an NMP/DMSO (70:30) mixture. Here, the addition of ethanol is key. It removes excess NaBH_4_ through ethanolysis, a process catalyzed by the in situ formed M(0) species (see equations in Figure 1). Immediately afterward, a film was cast from the casting‐reaction solution and immersed in a water bath, inducing phase separation. This precipitated the dissolved PES into a porous support membrane, accompanied by integration of the dispersed nanoparticles into the polymer matrix. Finally, the membrane‐immobilized zerovalent metal particles were oxidized to metal oxides via a mild, corrosion‐based post‐treatment. This was achieved by incubating the freshly fabricated membranes in an aqueous pH 9 solution (0.01 mm NaOH) for 24 h at ambient conditions.

We conducted the as‐described *all‐in‐one* fabrication using salts (55 µmol per 1 g of casting solution) of all transition metals from V to Zn (**Tables** **1**; S1, Supporting Information).

**Table 1:** Thickness, porosity, metal content, metal loading, and metal utilization for all metal‐decorated membranes, along with the redox potential values for the reaction of metal precursors to M(0). All materials were characterized after the oxidative post‐treatment, and averages and standard deviations were obtained from three different samples.

Membrane	Thickness [µm]	Porosity [%]	Metal content [wt.%]^a)^	Metal loading [µg cm^−2^]^a)^	Metal utilization [%]^b)^	Redox potential [V vs SHE]^[^ ^38^ ^]^
*M_PES*	106 ± 9	79 ± 4	−	−	−	−
*M_V*	105 ± 2	78 ± 5	0	0	0	V^3+^: −0.84
*M_Cr*	110 ± 3	84 ± 2	1.86 ± 0.14	48 ± 4	98 ± 7	Cr^3+^: −0.74
*M_Mn*	103 ± 4	78 ± 2	1.80 ± 0.10	45 ± 1	89 ± 5	Mn^2+^: −1.18
*M_Fe*	101 ± 2	82 ± 3	1.95 ± 0.04	48 ± 1	95 ± 2	Fe^3+^: −0.04
*M_Co*	110 ± 2	84 ± 4	1.94 ± 0.05	50 ± 2	90 ± 2	Co^2+^: −0.28
*M_Ni*	105 ± 1	84 ± 4	2.15 ± 0.10	52 ± 3	99 ± 4	Ni^2+^: −0.26
*M_Cu*	109 ± 5	80 ± 1	2.27 ± 0.08	57 ± 2	98 ± 3	Cu^2+^: 0.34
*M_Zn*	114 ± 6	85 ± 2	1.89 ± 0.02	46 ± 2	78 ± 1	Zn^2+^: −0.76

^a)^ Determined via acid degradation of metal‐decorated membrane, followed by AAS.

^b)^ Metal utilization [%] = Metal in membrane matrix [mol]/Metal salt added to casting‐reaction solution [mol].

NaBH_4_ has a redox potential of −1.24 V versus SHE (standard hydrogen electrode),^[^ ^38^ ^]^ theoretically providing enough driving force for reduction of all employed metal precursor ions to M(0). However, as shown by the absence of vanadium in *M_V*, dissolved VCl_3_ was not converted by NaBH_4_ to solid V(0), resulting in its release into the water precipitation bath during membrane immobilization. While the reduction of V^3+^ to V(0) has a net redox potential of −0.84 V versus SHE, this process can occur in two separate steps: from V^3+^ to V^2+^ and from V^2+^ to V(0). The latter step has a potential of −1.13 V versus SHE, which is close to that of NaBH_4_. Consequently, NaBH_4_ may not be able to facilitate this final reduction step. In contrast, the *all‐in‐one* method achieved exceptionally high utilization for all first‐row transition metals from Cr to Ni (89–99%), with a slightly lower value of 78% for zinc. For both zinc and vanadium, we employed chloride salts (Table S1, Supporting Information), indicating that Cl^−^ contributed to their less efficient in situ reduction. This is in line with electrochemical series that shows a lower redox potential of metal chloride complexes compared to these with other anions.^[^ ^39^ ^]^

Additionally, all the metal‐decorated membranes exhibit high porosities, with values between 78–85% (Table 1). Capillary flow porometry also revealed consistent through‐pore diameter distributions across all materials (Section S2, Supporting Information). Together, these observations indicate that the different metal particles were immobilized into support membranes with a similarly accessible pore network.

### 2.2 Membrane Characterization

**Figure 2:**
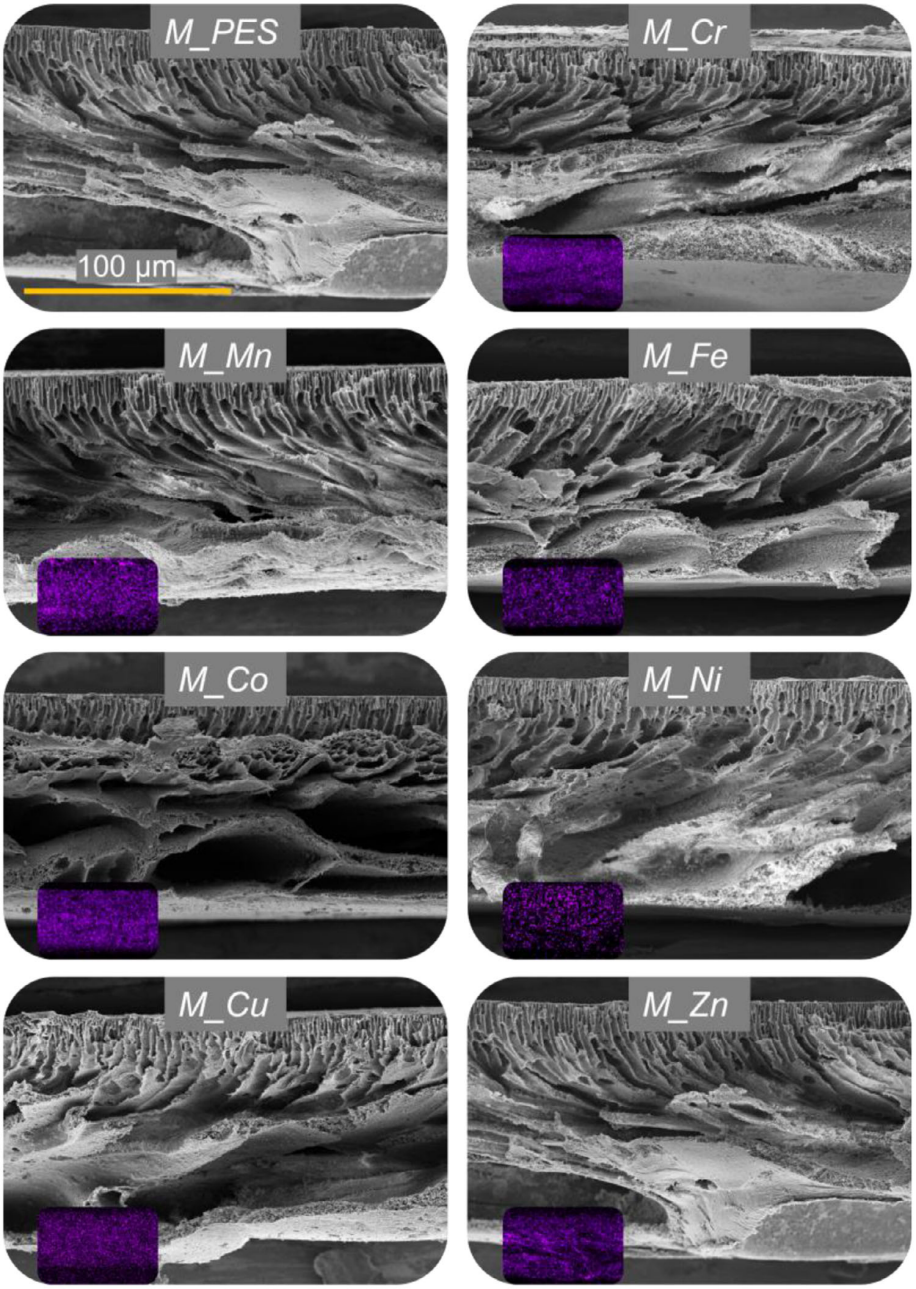
SEM images of the cross section of the metal‐decorated membranes. The EDX mapping of the corresponding transition metal across the displayed cross‐section is provided in the lower left corner of each image. The scale bar shown for *M_PES* applies to all SEM images.

We characterized the micro‐scale bulk properties of all metal‐decorated membranes (except *M_V)* via SEM imaging and EDX mapping (**Figure** **2**).

All materials exhibit an anisotropic finger‐like pore structure with a size gradient from small pore diameter in the upper region to larger pore diameters in the lower section. This structure results from the liquid non‐solvent induced phase separation (NIPS) that was employed to precipitate the matrix polymer after film casting. In this process, the top side of the cast film had contact to the water precipitation bath, which led to a gradual polymer solidification that generates a pore size gradient through the cross‐section.^[^ ^23^, ^40^ ^]^ Overall, the presence of solidified metals and products from the in situ reduction process did not significantly alter the pore morphology of the metal‐decorated membranes compared to a reference PES membrane (*M_PES*). Additionally, the EDX mapping demonstrates the successful immobilization of the different transition metals into the support membrane, and their excellent dispersion through the whole cross‐section (Figure 2, inset in lower left corner of each SEM image).

### 2.3 Immobilized Metal Catalyst Characterization

**Figure 3:**
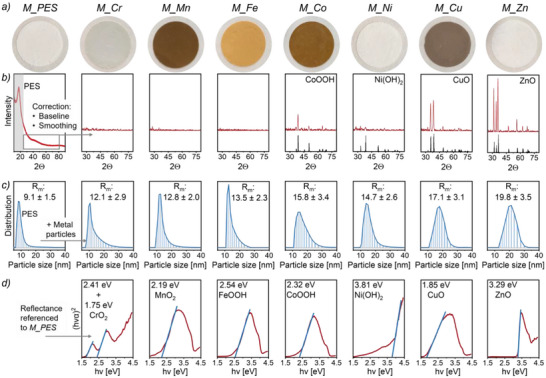
Characterization of membrane‐immobilized metal oxides. a) Pictures of metal‐decorated membrane coupons (20 mm). b) XRD profiles of metal‐decorated membranes (red) and reference XRD profiles (black). c) Particle size distribution determined via DLS after dissolving the support membrane in NMP. D) Optical bandgap energy of the metal oxides.^[^ ^41^, ^42^, ^43^, ^44^, ^45^, ^46^ ^]^ Tauc plots were generated from UV–vis reflectance spectra of metal‐decorated membranes that were referenced to *M_PES*.

Directly after fabrication, all membrane‐immobilized metal particles underwent a corrosion‐based post‐treatment in water at pH 9 for 24 h (Section S1, Supporting Information). Over the course of this treatment, the membranes changed their color from dark metallic (initial M(0) state) to a distinct, characteristic one (cf. Figure 1). To characterize the formed metal oxides, we further determined their XRD profiles, their particle sizes and their optical bandgaps (**Figure** **3**).

Rietveld refinement of the high‐intensity XRD signals of the *M_Co*, *M_Cu*, and *M_Zn* membranes yielded crystallite sizes of 15 nm for the immobilized cobalt, 14 nm for copper, and 23 nm for zinc (Section S3, Supporting Information). In contrast, for *M_Ni*, we observed only a low XRD signal intensity and no signals at all for the other metal oxide‐containing membranes (Figure 3b). The absence of XRD signals for some of the formed oxides is likely attributable to the low metal loading of the support membrane (cf. Table 1). Consequently, only metal species with strong scattering characteristics may be detectable.

The size of immobilized particles was further determined via DLS, after first dissolving the supporting membrane in NMP (Figure 3c). For the reference membrane *M_PES*, this procedure yielded an apparent mean radius (*R*_m_) of 9.1 nm, which matches the solvated radius of gyration reported in literature for the 72 kDa PES we used as matrix polymer (Section S1.1, Supporting Information).^[^ ^47^ ^]^ Notably, DLS analysis of the NMP‐dissolved metal‐decorated membranes similarly revealed a single population peak. This shows that the metal nanoparticles remained entangled with PES after dissolution, indicating that physically bonded metal‐polymer clusters formed during in situ particle synthesis in the casting solution. This could also explain the exceptional metal utilization of the *all‐in‐one* method (cf. Table 1). During polymer precipitation in the water bath, PES chains strongly bound to the nanoparticles may act as anchors, securing them within the forming support membrane. Importantly, the apparent mean radii of the dispersed metal‐polymer clusters ranged only between 12.1 and 19.8 nm across all metal species, illustrating that comparable particle sizes were obtained and that the *all‐in‐one* approach effectively mitigated irreversible agglomeration and sintering.

The XRD signals obtained for *M_Co*, *M_Ni*, *M_Cu*, and *M_Zn* identified the membrane‐immobilized metal species that formed after the oxidative post‐treatment as CoOOH, Ni(OH)_2_, CuO, and ZnO, respectively (Figure 3b). However, it should be noted that the peaks that were assigned to Ni(OH)_2_ are only slightly higher than the noise level. By comparing the determined bandgaps to literature values, the metal species inside the membranes *M_Cr*, *M_Mn*, and *M_Fe* can further be assigned to CrO_2_, MnO_2_, and FeOOH, respectively (Figure 3d).^[^ ^41^, ^42^, ^43^, ^44^, ^45^, ^46^ ^]^ We also performed electrochemical cell potential calculations to model the post‐treatment oxidation of zerovalent metals exposed to atmospheric oxygen in water at pH 9 (Section S4, Supporting Information).^[^ ^39^, ^48^ ^]^ The calculation results suggest a stepwise oxidation until the potential of dissolved oxygen can no longer drive any further reaction, with the thermodynamically favored oxide forming at the final metal oxidation state (Figure S2, Supporting Information). Importantly, the oxides predicted by this mechanism match those indicated by XRD and bandgap analysis, corroborating their identity.

Additionally, we did not observe XRD signals of any remaining M(0), indicating that the mild post‐treatment, using atmospheric oxygen at room‐temperature, fully oxidized all metals. This represents a significant achievement of the *all‐in‐one* platform, as other state‐of‐the‐art approaches often rely on hydrothermal or calcination methods with high energy demands to generate metal oxides.^[^ ^20^ ^]^ The fact that oxidation is a surface‐limited process, occurring only to depths in the low tens of nm for dense metals,^[^ ^49^ ^]^ further implies that the metal nanoparticles remain highly accessible following immobilization into the support membrane.

### 2.4 Benchmarking of Catalytic AOPs

We employed batch reactions for benchmarking the transition metal oxides in catalytic AOPs, because this configuration minimizes the performance variables in comparison to more practical variants like semi‐batch setups, fluidized batch reactors or catalytic membrane filtration. Comparisons between conventional powder catalysts can be limited by variations in their particle agglomeration level and re‐dispersion efficiency. Our membrane‐immobilized metal oxides circumvent this issue, because their use ensures that the catalyst particles are in a dispersed state during reaction. We tested all metal oxides as catalysts in the batch oxidation of tetracycline (TC), diclofenac (DF), cefalexin (CFX) and *p*‐nitrophenol (*p*‐NP) in water using persulfate (PS, S_2_O_8_^2−^), H_2_O_2_ and sulfite (SO_3_^2−^) as oxidants. To monitor the oxidation process of the different organic pollutants we investigated the conversion degree, the formation of colored degradation products, the oxidation degree of aromatic moieties, and the total organic carbon (TOC) removal (Figures S3–S6, Supporting Information). Of these, we found the aromatic oxidation degree to be the most reliable metric for assessing the extent of catalytic pollutant removal (Section S5, Supporting Information).

We further tested all catalyst‐oxidant‐pollutant combinations in presence of NaCl and NaHCO_3_ (as reference, PS was also tested with all catalyst‐pollutant combinations without these additional ions). Cl^−^ and HCO_3_^−^ are the most potent radical scavengers among ions commonly found in water.^[^ ^50^ ^]^ Furthermore, bicarbonate and carbonate can passivate metal oxide catalysts. Both these issues highlight the critical need to elucidate the reasons for performance variations between metal oxides under these conditions. It is also important to highlight that the goal of this work was not to develop a practical water treatment process, but to gain fundamental insight into the factors governing effectiveness of metal oxides in catalytic AOPs. Therefore, we specifically designed the experiments to yield incomplete removal degrees by using high pollutant concentrations (50 mg L^−1^), which is essential for comparing catalytic materials.^[^ ^51^ ^]^ We also exemplarily tracked the pollutant oxidation process over time using *M_Co* as catalyst and the combination of DF and PS (Section S6, Supporting Information). *M_Co* exhibited gradual DF removal, reaching 40% over 18 h, which indicates that the reaction was limited by catalyst activity rather than substrate availability under these conditions.

**Figure 4:**
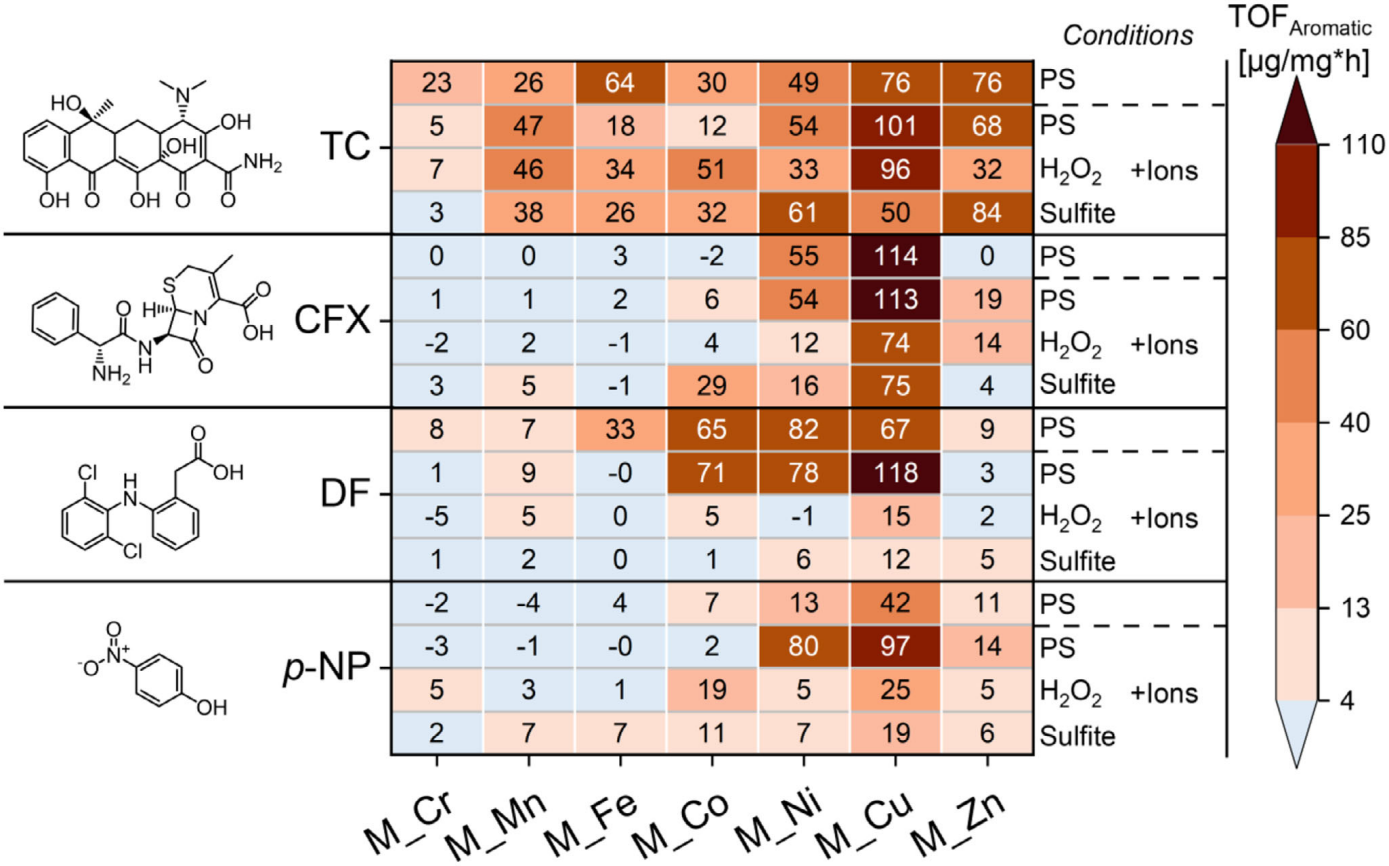
*TOF*_Aromatic_ of the different membrane‐immobilized metal oxides for the catalytic degradation of the aromatic moieties of TC, CFX, DF and p‐NP. PS, H_2_O_2_ and sulfite were used as oxidants, all in presence of NaCl and NaHCO_3_ as additional ions (pH 8). As reference, PS was also tested in absence of these ions (pH 7). All experiments with PS were conducted twice with different samples and the obtained averages are shown. Full experimental data can be found in Section S5 (Supporting Information). Conditions: 18 h reaction time, 30 °C, 10 mL, 50 mg L^−1^ organic pollutant, 5 mm oxidant, 5 or 0 mm NaHCO_3_, 3 or 0 mm NaCl, 3.14 cm^2^ membrane area, ≈0.16 mg metal in each sample.

To quantify performance, we calculated the turnover frequency (*TOF*_Aromatic_, µg degraded pollutant per mg metal per h) of all immobilized metal oxides for the oxidation of the aromatic moiety of the different organic pollutants (**Figure** **4**). To correct for pollutant adsorption onto the membrane matrix (Section S5, Supporting Information), we subtracted the aromatic removal degree of the inactive *M_PES* reference from that of the membrane‐immobilized metal oxides:

(1) $$\begin{array}{ccc} \mathrm{TO}F_{\mathrm{Aromatic}} & & =(\left(R_{\mathrm{Aromatic}}\;-\;R_{\mathrm{Aromatic}}\left(M\_\mathrm{PES}\right)\right)\;\times \;0.01\;\times \;m_{\mathrm{Pollutant}})\\ & & /\;(m_{\mathrm{Metal}}\;\times \;t_{\mathrm{Reaction}}) \end{array}$$

*R*_Aromatic_: aromatic removal degree of membrane‐immobilized metal oxide; *R*_Aromatic(M_PES)_: aromatic removal degree of M_PES (reference for impact of support membrane); *m*_Pollutant_: µg organic pollutant in reaction solution; *m*_Metal_: mg immobilized metal in sample; *t*_Reaction_: reaction time in h.

Averaging the *TOF*_Aromatic_ across all metal oxide catalysts (in presence of NaCl and NaHCO_3_) reveals the catalyst‐independent reactivity trends among the employed organic pollutants and oxidants. The average *TOF*_Aromatic_ decreased drastically from TC (43 ± 27 µg mg^−1^ h^−1^) to CFX (21 ± 31 µg mg^−1^ h^−1^), and then further to DF (16 ± 31 µg mg^−1^ h^−1^) and *p*‐NP (15 ± 25 µg mg^−1^ h^−1^) (Section S7, Supporting Information). This trend aligns with the electron density of their aromatic systems. The aromatic moiety in TC is relatively electron rich due to the +M effect of the OH group, which can promote oxidation. In contrast, CFX has no aromatic substituents, the two chlorides in DF have a ‐I effect, and the NO_2_ group of *p*‐NP is strongly electron‐withdrawing due to its ‐M effect. This suggests that electron density is a key factor impacting the susceptibility of organic pollutants for aromatic oxidation.

For the oxidants, the highest average *TOF*_Aromatic_ was achieved in presence of PS (35 ± 40 µg mg^−1^ h^−1^), which was significantly higher than with H_2_O_2_ (17 ± 24 µg mg^−1^ h^−1^) or sulfite (18 ± 23 µg mg^−1^ h^−1^). This average effectiveness of the oxidants across four pollutants and seven catalysts reveals intrinsic differences in their oxidative power, aligning with their respective redox potentials. Sulfite (SO_3_^2−^) has a negative potential (−0.68 V vs SHE at pH 7), and its oxidative power stems from the formation of the •SO_3_^−^ radical. Additionally, dissolved oxygen (0.76 V vs SHE) is often cited as a critical co‐oxidant in sulfite‐based AOPs, where it acts as the electron acceptor and sulfite as the electron donor in the radical‐generating catalytic cycle of metal oxides.^[^ ^52^ ^]^ For comparison, the redox potentials of H_2_O_2_ and PS at pH 7 are ≈1.3 and 2.0 V, respectively.^[^ ^38^ ^]^

**Figure 5:**
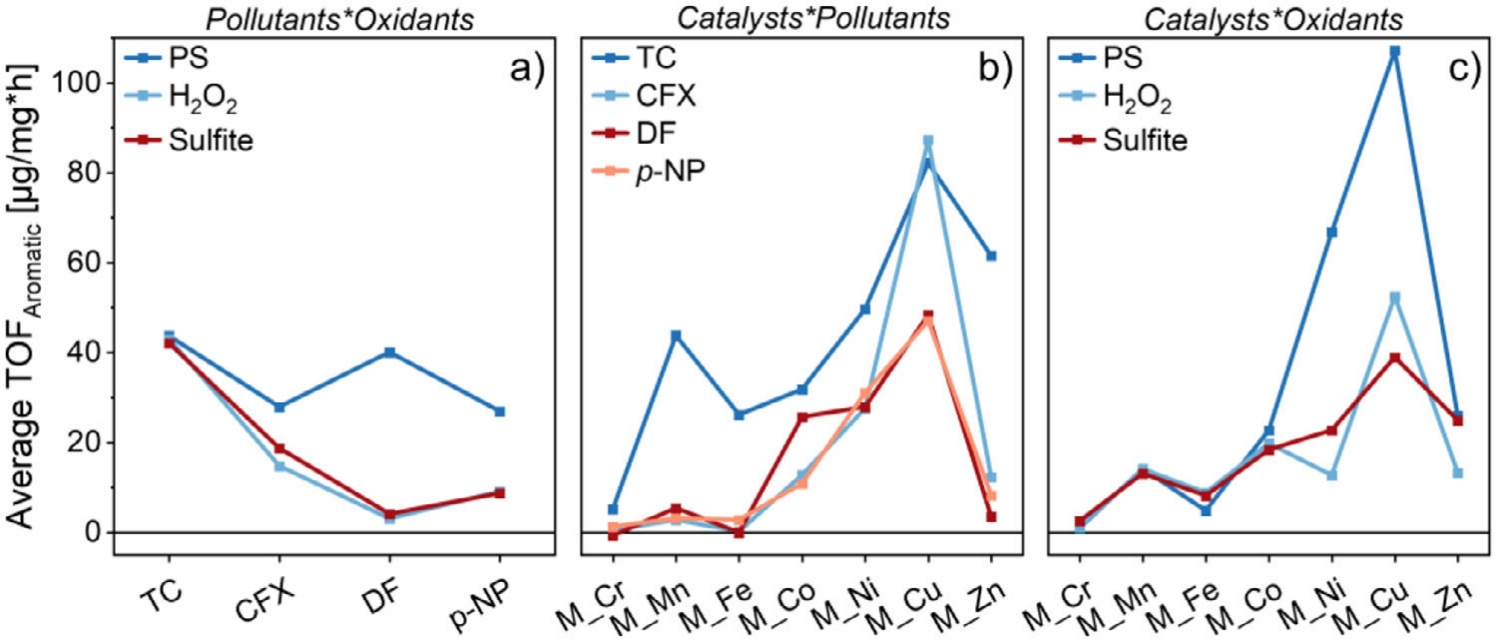
Average *TOF*_Aromatic_ values for the different combinations of pollutants and oxidants (a), catalysts and pollutants (b), and catalysts and oxidants (c). These values were calculated from benchmarking data across all experiments with NaCl and NaHCO_3_ (cf. Figure 4). For better visibility, the standard deviations of the average *TOF*_Aromatic_ values are omitted (Sections S5 and S7, Supporting Information).

To further elucidate how specific interactions contribute to these trends, we calculated the average *TOF*_Aromatic_ values (across all experiments in presence of NaCl and NaHCO_3_, cf. Figure 4) for each binary combination in the catalyst‐oxidant‐pollutant system (**Figure** **5**).

When averaged across all metal oxides, TC was catalytically oxidized with all three oxidants (Figure 5a). However, only PS efficiently oxidized the aromatic moieties of the other pollutants, while H_2_O_2_ and sulfite exhibited moderate oxidation of the electron‐neutral CFX and little degradation of the electron‐deficient DF and *p*‐NP. This trend matches the thermodynamic stability of aromatic compounds reported in literature, with redox potentials of >1.5 V versus SHE for electron‐deficient aromatics and between 0.9–1.4 V versus SHE for electron‐rich ones.^[^ ^53^ ^]^ A key implication of using high concentrations of Cl^−^ (3 mm) and HCO_3_^−^ (5 mm) is that primary radicals (•SO_4_^−^, •OH, •SO_3_^−^) are rapidly converted into the same secondary chloride and carbonate radicals in all systems, which have been shown to be the dominant reactive species under these conditions.^[^ ^54^, ^55^ ^]^ However, oxidation is only thermodynamically favored when the concentration‐dependent redox potential of the radicals exceeds that of the target pollutant, making their concentration a crucial factor. This suggests that each oxidant possesses intrinsic properties that dictate the catalytic radical generation rate across all metal oxides. A plausible explanation is that the oxidant's redox potential drives the electron transfer from a metal site to the oxidant, thereby initiating and sustaining the radical‐generating catalytic cycle (see Section 2.6 for a detailed theoretical examination of the catalytic mechanism). Consequently, a higher oxidant redox potential (e.g., for PS) should enhance the radical generation rate, producing a concentration sufficient to oxidize even electron‐deficient aromatic compounds.

In contrast, we observed that metal oxide activity is not generally dependent on specific catalyst–pollutant interactions (Figure 5b). The average *TOF*_Aromatic_ values of the different combinations of organic pollutants and catalysts match the proposed pollutant reactivities, and the activity trend among the metal oxides is similar across all pollutants (Figure 5b). The only exception is the combination of *M_Cu* and CFX, for which we observed the highest *TOF*_Aromatic_ across all experiments. CFX contains a thioether group (cf. Figure 4), which may enhance pollutant adsorption onto CuO due to the specific affinity of copper for thio‐groups.

Furthermore, the *TOF*_Aromatic_ values resulting from the different combinations of metal oxides and oxidants illustrate a distinct activity pattern for each oxidant, again demonstrating the importance of oxidant–catalyst interactions (Figure 5c). However, with all oxidants an increasing activity trend from *M_Cr* to *M_Cu*, followed by a decline toward *M_Zn*, can also be observed. This indicates that each metal oxide has an intrinsic activity for oxidant activation, which is subsequently influenced by specific interactions between the chosen oxidant and the catalyst. The alignment of the intrinsic activity trend with the increasing atomic number of the involved transition metals further suggests a contribution of their electronic properties.

### 2.5 Impact of Dissolved Cl^−^ and HCO_3_^−^

**Figure 6:**
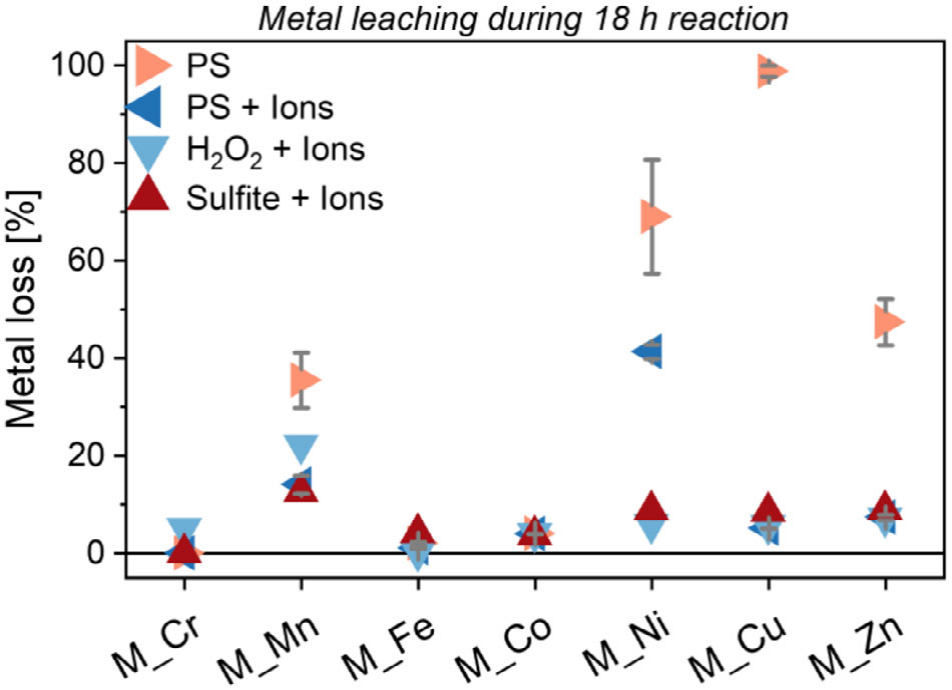
Relative metal loss from the supporting membrane during 18 h reaction in presence of different oxidants and with TC as organic pollutant (same experiments as shown in Figure 4). All experiments with PS as oxidant were conducted twice with different membrane samples and the obtained averages and standard deviations are shown.

Next, we analyzed the impact of NaCl and NaHCO_3_ as additional ions on the catalytic AOP performance. For this, we quantified the amount of metal cations that leached from the membrane‐immobilized metal oxides into the reaction solution during the benchmarking experiments with TC (**Figure** **6**). Due to the employed reaction time of 18 h, this gives insights into the general stability of the different metal species.

For *M_Mn*, *M_Ni*, *M_Cu*, and *M_Zn*, we observed strong metal cation leaching in the presence of PS alone. However, the addition of NaCl and NaHCO_3_ almost completely mitigated PS‐facilitated metal loss from the supporting membrane except for *M_Ni*, and in their presence neither H_2_O_2_ nor sulfite caused extensive metal leaching. This suggests that additional ions in water can interfere with the catalyst–oxidant surface interaction, thereby reducing the formation of soluble metal cation‐oxidant complexes. Metal complexation behavior can be described by chemical hardness (*η*), as proposed by Pearson and Parr.^[^ ^56^ ^]^ *η* quantifies a compound's resistance to changes in its electron density distribution, such as those occurring during interactions between an electron density donor (Lewis base) and an electron density acceptor (Lewis acid). For solid semiconductors, *η* is defined as half the bandgap energy, reflecting the energy barrier for redistributing bound electrons. In a Lewis acid–base pair (e.g., S_2_O_8_^2−^, a base, interacting with a surface‐bound metal cation as acid), the binding affinity is generally stronger when both partners have either low (“soft”) or high (“hard”) *η* values.

**Figure 7:**
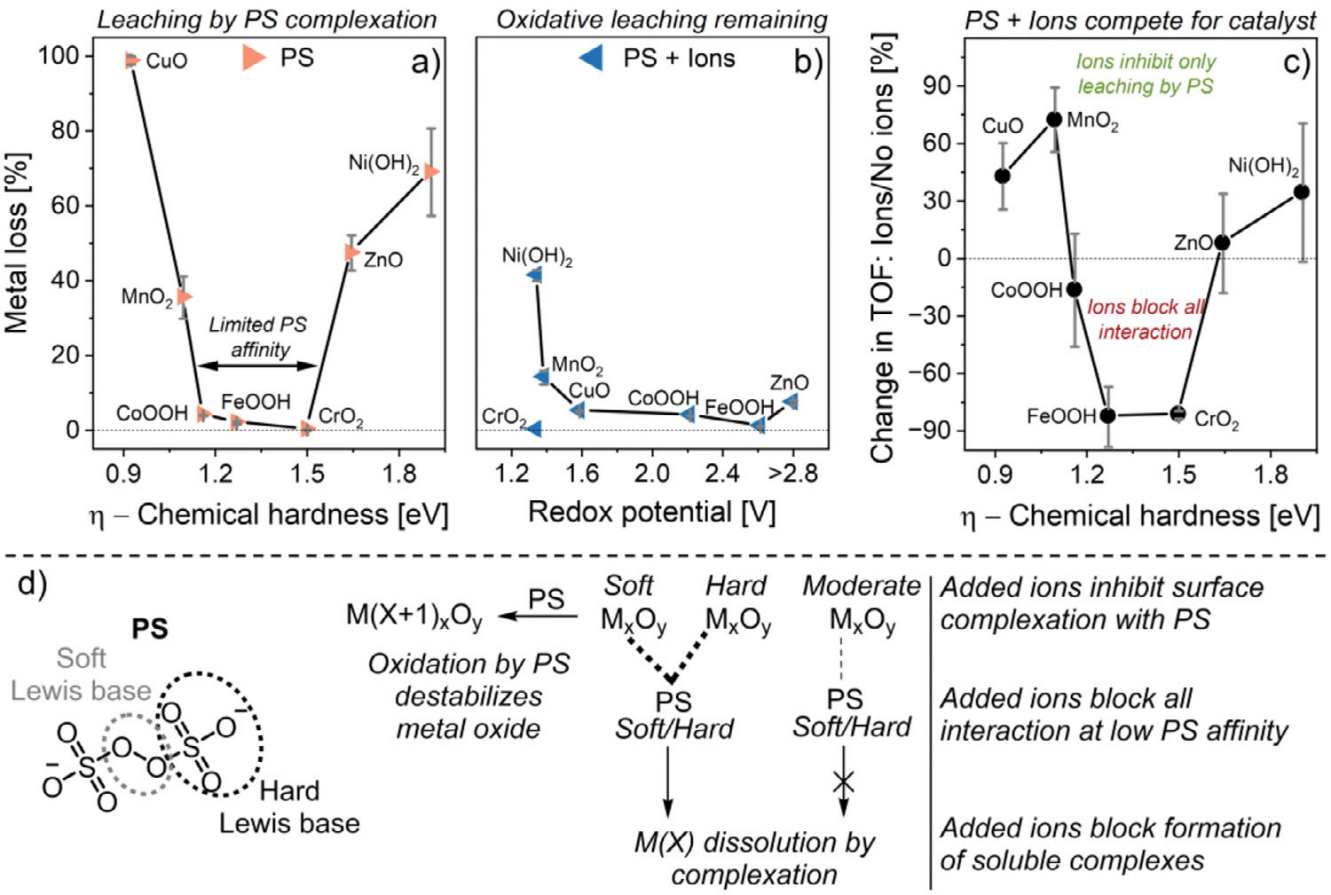
a) Chemical hardness of metal oxides in relation to their metal loss during 18 h reaction with PS alone. b) Redox potential of metal oxides in relation to their metal loss during 18 h reaction with PS in presence of NaHCO_3_ (5 mm) and NaCl (3 mm). ZnO cannot be further oxidized to another oxide species, but its redox potential is shown as “>2.8 V” for better visualization. c) Chemical hardness of metal oxides in relation to the relative change in their *TOF*_Aromatic_ with PS as oxidant, caused by addition of NaHCO_3_ and NaCl. The shown averages and standard deviations were calculated from the data obtained with all four organic pollutants (cf. Figure 4). d) Proposed mechanism for interaction between PS and metal oxide surfaces.

We calculated *η* for all the membrane‐immobilized metal oxides from their optical bandgaps (cf. Figure 3), and analyzed the influence of their chemical hardness on catalyst stability and activity (**Figure** **7**). For CrO_2_ (*M_Cr*), we used a theoretical *η* value of 1.5 eV from literature because CrO_2_ is half‐metallic, meaning the optical bandgap does not directly relate to its actual bandgap.^[^ ^57^ ^]^

This analysis reveals that metal cations were leached by PS only from the membrane‐immobilized metal oxides that exhibit either low or high chemical hardness, with no leaching observed for species with moderate η values (Figure 7a). PS (S_2_O_8_^2−^) can coordinate to metal oxide surfaces either via its peroxo or sulfate groups, with the former likely acting as soft Lewis base (non‐charged and low electron density) and the latter as hard Lewis base (charged and high electron density). This means PS may only effectively interact with metal oxides that have either low or high chemical hardness and, consequently, may only facilitate metal cation leaching from these metal oxides (Figure 7d). However, the addition of NaHCO_3_ and NaCl mitigated this metal loss, possibly due to HCO_3_^−^ competing with PS for metal cation complexation, only that complexes with HCO_3_^−^ are usually water‐insoluble. Nevertheless, some residual PS‐facilitated metal loss still remained in presence of NaHCO_3_ and NaCl, and we found that the amount correlates with the redox potential of the membrane‐immobilized metal oxides (Figure 7b, redox potential for formation of the metal oxide with the next higher metal oxidation state^[^ ^39^ ^]^). This suggests that a lower redox potential facilitates oxidation of the bulk metal oxide, potentially leading to formation of a separate surface layer that is more susceptible to leaching. This oxidative leaching likely always occurs but is probably overshadowed by complexation leaching in the absence of NaHCO_3_ and NaCl. For CrO_2_, despite its low redox potential of 1.34 V versus SHE, we observed no oxidative leaching with PS. This is consistent with the proposed impact of the moderate chemical hardness of CrO_2_, which may prevent all forms of interaction with PS.

Similarly, the average *TOF*_Aromatic_ of metal oxides with moderate chemical hardness decreased by up to 90% across all four pollutants following addition of HCO_3_^−^ and Cl^−^ to PS (Figure 7c). This further supports that their affinity for PS is limited and, therefore, PS cannot compete with other anions. In contrast, metal oxides with low and high *η* values exhibited increases in *TOF*_Aromatic_. First, this highlights an effective catalyst–PS interaction even at relatively high concentrations of interfering anions in water. Second, the enhanced catalytic activity with NaHCO_3_ and NaCl is likely due to the mitigation of PS‐facilitated metal loss, a process that was most severe for the PS‐affine metal oxides with low and high η values.

### 2.6 Framework for Predicting Activity of Metal Oxides

Based on these experimental observations, we developed a theoretical framework connecting the physicochemical properties of metal oxides with their catalytic function in AOPs.

**Figure 8:**
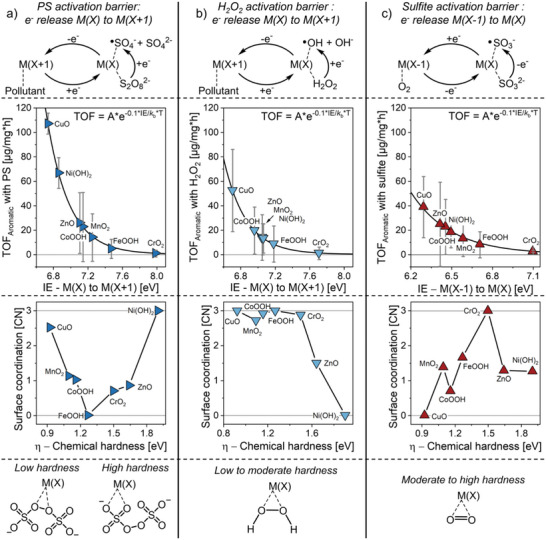
Schematic oxidant activation mechanism, *TOF*_Aromatic_ of metal oxides (average and standard deviation were calculated from all experiments with NaCl and NaHCO_3_, cf. Figure 4) in relation to their modeled oxidant‐specific *IE*_MAS_, predicted effective surface coordination of MAS during the electron release step of the catalytic redox cycle in relation to their chemical hardness (Section S9, Supporting Information), and schematic catalyst‐oxidant complexation behavior. a) PS (S_2_O_8_^2−^). b) H_2_O_2_. c) Sulfite (SO_3_^2−^).

The proposed framework is characterized as follows (see Section S8 in the Supporting Information for more details):

- The reaction between pollutants and generated radicals is assumed to be fast, thereby making oxidant activation rate‐determining.
- Oxidants are activated by metal active sites (MAS) through a catalytic redox cycle (**Figure** **8**), in which MAS switch between oxidation states via one‐electron transfers while generating radicals.
- Activation of PS and H_2_O_2_ involves a M(X) → M(X+1) → M(X) transition. In contrast, sulfite activation involves a M(X) → M(X‐1) → M(X) transition due to its negative redox potential (with dissolved oxygen facilitating the electron release as co‐oxidant).
- We propose that electron release from M(X) or M(X‐1) constitutes the rate‐determining step of the catalytic redox cycle, driven by the redox potential of the oxidant. Consequently, the ionization energy (IE) of MAS corresponds to the energy barrier for electron release and can serve as a descriptor for the activation energy of catalytic radical generation.
- We propose that MAS can be described as metal cations bound at the bulk metal oxide surface. Consequently, the IE of an individual MAS should result from the electronic properties of the metal cation species that constitutes the MAS, as well as from the bulk electronic properties of the metal oxide the MAS is bound to.

In this framework, the electronic properties of the metal cation species that constitute the MAS are described through their Mulliken electronegativity as follows:

(2) $$X_{M}=0.5\times \left(\mathrm{IE}+\mathrm{EA}\right)$$

*Χ*_M_: Mulliken electronegativity of metal cation [eV]; IE: ionization energy [eV]; EA: electron affinity [eV].

The electronic properties of the bulk metal oxide correspond to their band structure, which can be described by the bandgap energy as follows:

(3) $$E_{g}=\mathrm{VB}\;-\;\mathrm{CB}=\mathrm{IE}\;-\;\mathrm{EA}$$

*E*_g_: bandgap energy [eV]; VB: valence band energy [eV]; CB: conduction band energy [eV] IE: ionization energy [eV]; EA: electron affinity [eV].

Following the assumption that the IE of MAS results from the electronic properties of both the metal cation species and the bulk metal oxide, we propose that their IE can be approximated as follows:

(4) $${\mathrm{IE}}_{\mathrm{MAS}}=X_{M}+0.5\times E_{g}$$

*IE*_MAS_: ionization energy of MAS [eV]; *Χ*_M_: Mulliken electronegativity of metal cation [eV]; *E*_g_: bandgap energy of the bulk metal oxide [eV].

For calculating the Mulliken electronegativity of metal cation species, an empirical relationship established by Matar et al. was utilized (Equation S3, Supporting Information),^[^ ^57^ ^]^ in which *Χ*_M_ is impacted by the metal cation properties as follows:

(5) $$X_{M}\approx Z_{M}$$

*Χ*_M_: Mulliken electronegativity of metal cation; *z*_M_: charge number of metal cation.

And:

(6) $$X_{M}\;\approx \;1/r_{M}$$

*Χ*_M_: Mulliken electronegativity of metal cation; *r*_M_: ionic radius of metal cation.

Overall, our framework predicts that the intrinsic *IE*_MAS_ of metal oxides (intrinsic activity for redox cycling) decreases when its metal sites are in a lower oxidation state, have a higher ionic radius, and the bulk material has a lower bandgap energy. For a known metal oxide in an isolated state, this intrinsic *IE*_MAS_ can be directly calculated, as the charge number and ionic radius of the metal sites are defined by the crystal structure and oxide type. However, when the metal oxide exists in a reaction solution, the surface coordination of MAS with molecules can influence their ionic radius. This relationship can be described as follows:

(7) $$CN_{\mathrm{MAS}}\;\approx \;r_{M}\;\approx \;1/\;\;\;X_{M}$$

*CN*_MAS_: surface coordination number of MAS; *r*_M_: ionic radius of metal cation; *Χ*_M_: Mulliken electronegativity of metal cation.

With an increasing surface coordination number (*CN*), the ionic radius of MAS is expected to increase, thereby reducing their *Χ*_M_. Consequently, this framework predicts that the intrinsic *IE*_MAS_ of metal oxides dynamically changes when MAS interact with electron‐acceptor molecules during electron release. However, the resulting oxidant‐specific *IE*_MAS_ cannot be directly calculated, as the surface *CN* of MAS during the reaction is unknown.

Therefore, we propose a modified Arrhenius equation, connecting the oxidant‐specific *TOF*_Aromatic_ of a metal oxide to its oxidant‐specific *IE*_MAS_:

(8) $$\mathrm{TO}F_{\mathrm{Aromatic}}=A\times e^{\left(\mathrm{pf}\times -IE_{\mathrm{MAS}}\right)/\left(k_{b}\;\times \;T\right)}$$

*TOF*_Aromatic_: oxidant‐specific average *TOF*_Aromatic_ of the metal oxide [µg mg^−1^ h^−1^]; A: maximum catalytic *TOF*_Aromatic_ achievable with the employed oxidant under the investigated reaction conditions [µg mg^−1^ h^−1^]; pf: proportionality factor, estimated as 0.1, which converts IE values to the order of magnitude commonly observed for activation energies; *IE*_MAS_: oxidant‐specific *IE*_MAS_ of the employed metal oxide [eV]; *k*_b_: Boltzmann constant [eV K^−1^]; T: Reaction temperature [K].

Based on Equation 8, we translated this mechanistic framework into a mathematical procedure for modeling the oxidant‐specific *IE*_MAS_ of the investigated metal oxides (Sections S8 and S9, Supporting Information) (Figure 8, the shown *TOF*_Aromatic_ values are averages calculated for each catalyst‐oxidant combination across all four pollutants).

This procedure successfully produced oxidant‐specific *IE*_MAS_ values for all metal oxides that are within the physical boundaries of the developed framework, and that match their observed *TOF*_Aromatic_ values (Tables S3–S6, Supporting Information). This implies that the proposed assumptions (i.e., rate‐determining oxidant activation, the redox cycle mechanisms, and the nature of MAS) accurately describe the pollutant degradation by the investigated metal oxides under the employed conditions. Consequently, the high *TOF*_Aromatic_ values observed for CuO, Ni(OH)_2_, and ZnO can be attributed to their low intrinsic *IE*_MAS_, stemming from their low metal oxidation state compared to the other metal oxides (cf. Equation 5). Additionally, the superior catalytic performance of CuO can be ascribed to the impact of its low bandgap energy (cf. Figure 3), which further decreases its intrinsic *IE*_MAS_ (cf. Equation 4).

Moreover, we extracted the effective surface *CN* of MAS from the modeled oxidant‐specific *IE*_MAS_ values, where a higher *CN* reflects a lower oxidant‐specific *IE*_MAS_ compared to the intrinsic one (Section S8, Supporting Information). For PS as oxidant (Figure 8a), the predicted surface *CN* increases for metal oxides with both low and high chemical hardness. This matches the impact of chemical hardness on PS‐facilitated metal cation leaching we observed (cf. Figure 7), supporting the hypothesis that PS binds efficiently to the surface of metal oxides when their hardness aligns with either the soft or hard functional groups of PS. For H_2_O_2_ as oxidant (Figure 8b), this analysis yielded continuously decreasing surface *CN* of MAS with increasing chemical hardness of the metal oxide. This aligns with the relative softness of the peroxo moiety as a Lewis base. This may also explain why Ni(OH)_2_, with high chemical hardness, is a significantly less effective catalyst for H_2_O_2_ activation (ranking fifth among all investigated metal oxides) compared to PS (ranking second), despite both oxidants being activated through the same catalytic redox cycle. In contrast, sulfite activation differs from that of PS and H_2_O_2_ because sulfite likely produces M(X‐1) active sites due to its negative redox potential, and, therefore, is not involved in the rate‐determining electron release from MAS. Consequently, electron release occurs to another acceptor, which under the conditions investigated here is most likely dissolved O_2_. O_2_ has a moderate chemical hardness,^[^ ^56^ ^]^ which matches the predicted surface *CN* of MAS during the electron release step of sulfite activation (Figure 8c). CrO_2_, with a chemical hardness of 1.5 eV, shows the highest *CN*, while it declines for metal oxides with both lower and higher hardness.

We also analyzed the impact of variations in *TOF*_Aromatic_ on the modeling of *IE*_MAS_ and *CN* values, as discussed in Section S10 (Supporting Information). This analysis shows both the general robustness and specific limitations of our approach. Furthermore, the framework implies that catalytic activity is highest at low Mulliken electronegativity, which corresponds to metal sites in their lowest possible oxidation state (cf. Equations 4 and 5). The Mulliken electronegativity of M(II) sites increases in the following order: Mn < Ni < Cr < Zn < Co < Fe < Cu. Consequently, incorporating these M(II) sites into a stabilizing bulk oxide, such as Fe_2_O_3_ or FeOOH, should increase catalytic activity relative to both the undoped bulk oxide and the high‐valent single oxides (e.g., MnO_2_ with Mn(IV)). As detailed in Section S11 (Supporting Information), we used the *all‐in‐one* method to synthesize membrane‐immobilized FeOOH catalysts doped with various secondary metals (Mn, Co, Ni, Cu, Zn). Their experimentally determined activities in both DI water and a complex ionic matrix (Table S8, Supporting Information) are in good agreement with the predictions of the framework (Figures S13 and S14, Supporting Information). Relative to pure FeOOH, doping with Mn and Ni enhanced the PS activation rate by up to ten and fivefold, respectively. This finding is consistent with the high activity reported in literature for MnFe_2_O_4_ and NiFe_2_O_4_.^[^ ^58^, ^59^ ^]^ Crucially, our mathematical modeling procedure was able to describe the catalytic activity in the complex ionic matrix. This further supports that the framework captures not only electron transfer but also surface adsorption processes, potentially enabling the modeling of catalytic performance of metal oxides under variable AOP conditions.

## 3 Conclusion

We established an *all‐in‐one* platform for the synthesis of membrane‐immobilized metal oxides composed of Cr, Mn, Fe, Co, Ni, Cu, and Zn. This method achieves near‐perfect metal utilization, enables preparation of different catalysts under identical conditions, mitigates particle agglomeration, eliminates separation and purification steps, and circumvents high‐temperature treatment. These characteristics represent a substantial improvement compared to other state‐of‐the‐art synthesis approaches. This *all‐in‐one* technique could also allow for the straightforward upscaling of immobilized metal oxide preparation by leveraging established roll‐to‐roll film casting processes. Beyond the specific metal species obtained in this study, modifying the post‐treatment oxidation protocol to incorporate the known effects of corrosion conditions on metal oxidation could further unlock access to diverse or specific metal oxide species.

We benchmarked the specific activity of the different metal oxides across various catalytic AOPs. CuO was found to be the best‐performing catalyst among those prepared, demonstrating high pollutant degradation rates under application‐relevant conditions (near‐neutral pH and in the presence of interfering anions). This performance is notable because many metal oxides reported in the literature exhibit significantly lower catalytic activities under those conditions than the here presented CuO.^[^ ^60^, ^61^, ^62^ ^]^ This can likely be ascribed to the *all‐in‐one* approach, which immobilized the CuO nanoparticles directly after their synthesis in a porous matrix. This mitigated irreversible agglomeration and ensured that all nanoparticles are in a dispersed state during reaction, which is often not the case when using powder catalysts. Furthermore, the high performance of the membrane‐immobilized CuO also aligns with recent reports which indicate that confinement can enhance catalytic activity of metal oxides.^[^ ^63^ ^]^

We also elucidated how the oxidant affinity and redox potential of metal oxides influence their resistance to interference from dissolved anions in water during AOPs, as well as their susceptibility to metal cation leaching. In addition, we developed a novel theoretical framework that explains how specific material properties of metal oxides govern their oxidant activation behavior, along with a mathematical modeling procedure to predict their activity under various reaction conditions. These new insights provide guidelines for designing metal oxides with potentially improved performance and long‐term stability in AOPs, addressing the most critical limitations currently hindering wider adoption of catalytic water treatment technologies.

## 4 Experimental Section

A detailed description of the experimental procedures can be found in Section S1 (Supporting Information).

## Conflict of Interest

The authors declare no conflict of interest.

## Supporting information



Supporting Information

## Data Availability

The data that support the findings of this study are available from the corresponding author upon reasonable request.
