# Supplementary material for: Material‐Efficient Synthesis of Polymer Membrane‐Immobilized Metal Oxides for Advanced Oxidation Processes: Catalytic Mechanism, Stability and Performance
Source: Small. 2025 Aug 15;21(39):e07532. doi: 10.1002/smll.202507532 (PMC12490189; doi:10.1002/smll.202507532)
Supplement: Supplementary file 1 — Supporting Information [file SMLL-21-e07532-s001.docx]

Supporting Information

Material-efficient synthesis of polymer membrane-immobilized metal oxides for advanced oxidation processes: Catalytic mechanism, stability and performance

Amir H. Hesaraki^a^, Burcu Önal^a^, Oleg Prymak^b,c^, Zhijun Ren^d^, Mathias Ulbricht^a,c^, Lukas Fischer^a,c,*^

^a^Lehrstuhl für Technische Chemie II and Center for Water and Environmental Research (ZWU), Universität Duisburg-Essen, Universitätsstr. 7, 45141 Essen, Germany

^b^Inorganic Chemistry, University Duisburg-Essen, Universitätsstr. 5, 45141 Essen, Germany

^c^Center for Nanointegration Duisburg-Essen (CENIDE), University Duisburg-Essen, Carl-Benz-Str. 199, 47057 Duisburg, Germany

^d^Tianjin Key Laboratory of Clean Energy and Pollutant Control, School of Energy and Environmental Engineering, Hebei University of Technology, Tianjin 300401, China

*Corresponding author.

Email: Lukas.Fischer@uni-due.de

S1. Experimental procedures

S1.1 Materials

Polyethersulfone (PES) Ultrason® 6020P was obtained from BASF. Dimethyl sulfoxide (DMSO) (99%), N‑methyl-2-pyrrolidone (NMP) (99%), NaBH_4_ (99%), K_2_S_2_O_8_ (99%), tetracycline (TC) (>98%), diclofenac sodium salt (DF) (>98%), *p*-nitrophenol (*p*-NP) (99%), VCl_3_ (99%), Cr(NO_3_)_3_*9H_2_O (99%), Mn(NO_3_)_2_*H_2_O (>98%), FeCl_3_*6H_2_O (>97%), Co(NO_3_)_2_*6H_2_O (>98%) and Ni(NO_3_)_2_*6H_2_O (>97%) were bought from Sigma Aldrich Co. NaHCO_3_ (99.5%) and NaCl (99.5%) were obtained from Thermo Fisher Scientific. Na_2_SO_3_ (>98%), cefalexin hydrate (CFX) (>97%) and ZnCl_2_ (>98%) were bought from Fluka. Cu(NO_3_)_2_*3H_2_O (>98%) was obtained from abcr GmbH.

S1.2 Synthesis of polymer membrane-immobilized transition metal oxides

15 wt% PES was dissolved in a 70/30 mixture of NMP/DMSO. Then, 55 µmol metal salt was added per 150 mg PES, respectively per 1 g of casting-reaction solution, followed by shaking overnight (Table S1).

Afterward, NaBH_4_ (1 wt%, ~5 mol eq. to metal) and ethanol (5 wt%) were added and the casting-reaction solution was high-shear mixed for 1 min (neoLab 7 2020 vortex mixer at 3150 rpm). The *in situ* metal reduction was then conducted in the casting-reaction solution for 2 h under constant ultrasonication in a sonication bath at 30°C, 45 kHz and 100 W (Transonic TI-H-10 from Elma). Finally, the casting-reaction solution was degassed for 20 min in a vacuum oven at 50°C and 50 mbar.

The integrated porous membrane preparation and metal particle immobilization was conducted by casting the casting-reaction solution after *in situ* metal reduction on a glass plate with a film thickness of 200 µm and at a casting speed of 10 mm/s (at room-temperature of ~22°C and at ~30% humidity). For this, we used a COATMASTER film applicator and a fixed gap doctor blade (Model 360), both from Erichson. After casting, the film was directly immersed in a deionized (DI) water precipitation bath at room-temperature, leading to formation of the metal-decorated porous polymer membrane (alternatively called porous polymer membrane-immobilized metal particles) through liquid non-solvent induced phase separation (NIPS).

After fabrication, the membrane, containing zerovalent metal particles at this point, was placed in DI water with a pH of 9 (0.01 mM NaOH) for 24 hours as a room-temperature oxidative post-treatment. After post-treatment, all membranes were stored in that same solution and used for catalytic experiments without any further treatment.

*Table S1: Compositions of the casting-reaction solutions used for the all-in-one fabrication of porous metal-decorated polymer membranes (per 1 g solution).*

| Membrane | PES [mg] | NMP [mg] | DMSO [mg] | Ethanol [mg] | Metal salt | Metal [µmol] | NaBH_4_ [mg] |
| --- | --- | --- | --- | --- | --- | --- | --- |
| *M_PES* | 150 | 595 | 255 | 50 | - | - | - |
| *M_V* | 150 | 595 | 255 | 50 | VCl_3_ | 55 | 10 |
| *M_Cr* | 150 | 595 | 255 | 50 | Cr(NO_3_)_3_*9H_2_O | 55 | 10 |
| *M_Mn* | 150 | 595 | 255 | 50 | Mn(NO_3_)_2_*H_2_O | 55 | 10 |
| *M_Fe* | 150 | 595 | 255 | 50 | FeCl_3_*9H_2_O | 55 | 10 |
| *M_Co* | 150 | 595 | 255 | 50 | Co(NO_3_)_2_*6H_2_O | 55 | 10 |
| *M_Ni* | 150 | 595 | 255 | 50 | Ni(NO_3_)_2_*6H_2_O | 55 | 10 |
| *M_Cu* | 150 | 595 | 255 | 50 | Cu(NO_3_)_2_*3H_2_O | 55 | 10 |
| *M_Zn* | 150 | 595 | 255 | 50 | ZnCl_2_ | 55 | 10 |

S1.3 Material characterization

To determine the porosity, three 14 mm diameter samples of each membrane were first weighed in a water-wet state, then weighed again and measured for thickness in a dry state using a Digital Micrometer IP65 from Mitutoyo. The porosity was calculated as follows:

*ε = (m_w_−m_d_) / (V_M_) * 100 (S1)*

*ε: membrane porosity; m_w_: membrane weight in water-wet state; m_d_: membrane weight in dry state; V_m_: membrane volume.*

To determine the metal content, three 14 mm diameter samples of each membrane were separately degraded in 60% HNO_3_ for 7 days. The amount of metal that leached from the membranes into the acid solution was then quantified via atomic absorption spectroscopy (AAS) using a Thermo Scientific AA spectrometer M Series FS95.

For scanning electron microscopy (SEM) and energy-dispersive X-ray spectroscopy (EDX), membrane samples were first dried and then frozen in liquid nitrogen, followed by braking to access the cross section. Before analysis, the cross section was sputter coated with a PdAu alloy. SEM and EDX were conducted on an Apreo S LoVac system from Thermo Fisher Scientific at a pressure of 10^-3^ Pa, an accelerating voltage of 5 kV (SEM) or 20 kV (EDX), and a current of 13 pA.

For the X-ray diffraction (XRD) analysis, membrane coupons of 24 mm diameter were first dried at room-temperature, then fixated on a flat silicon single crystal sample holder, and the XRD profile was obtained with a Panalytical Empyrean instrument in reflection mode using Cu Kα radiation (λ = 1.54 Å; 40 kV, 40 mA). The membranes were investigated from 5 to 90° 2Θ with a step size of 0.006° and a scan speed of 0.024 °/s, resulting in a total measurement time of 60 min. Qualitative phase analysis was performed with Diffrac.Suite EVA V1.2 (Bruker) and Rietveld refinement was performed with TOPAS 5.0 (Bruker). For this, the instrumental parameters were determined with the internal standard lanthanum hexaboride (LaB6) from NIST.

For dynamic light scattering (DLS) analysis of the membrane-immobilized particles, a 14 mm diameter coupon of each membrane was dried at room-temperature. Then, the membrane coupons were dissolved in 5 mL NMP via ultrasonication in a sonication bath at 30°C, 45 kHz and 100 W (Transonic TI-H-10 from Elma). Afterward, the resulting dispersion of metal particles in PES in NMP was analyzed (30 s measurement time) using a NANO-flex® 180° DLS system from Particle Metrix GmbH.

To determine the optical band gap of the membrane-immobilized transition metal oxides, a 20 mm diameter coupon of each membrane was dried at room-temperature. Then, the relative diffuse reflectance (compared to a bare PES membrane) of the membrane coupons was determined in the wavelength range from 200 to 800 nm via the integrating sphere method using a UV-2600i system from Shimadzu. From the obtained reflectance data, a Tauc plot was generated for each membrane, from which the optical band gap of the incorporated metal oxide was determined.

S1.4 Catalytic advanced oxidation processes (AOPs)

For the investigation of the catalytic performance of the fabricated porous membrane-immobilized transition metal oxides, we employed four different organic pollutants: tetracycline (TC), diclofenac (DF), cefalexin (CFX), and *p*-nitrophenol (*p*-NP), each at a concentration of 50 mg/L in the reaction solution. We further employed three different oxidants: persulfate (PS, S_2_O_8_^2-^), hydrogen peroxide (H_2_O_2_), and sulfite (SO_3_^2-^), each at a concentration of 5 mM in the reaction solution. Additionally, to test the impact of dissolved ions on catalytic performance, reaction solutions that additionally contained 5 mM NaHCO_3_ and 3 mM NaCl were employed.

All membranes were tested with each combination of organic pollutant and oxidant in presence of NaHCO_3_ and NaCl. Additionally, each membrane was tested with each organic pollutant in absence of NaHCO_3_ and NaCl using PS as oxidant. Overall, all fabricated membranes were tested in 16 different reaction conditions. For all experiments with PS as oxidant, two samples of each membrane were tested separately, and average values and standard deviations were calculated from the obtained data.

All reactions were conducted with 20 mm diameter membrane coupons immersed in 10 mL reaction solutions. The reaction solutions consisted of DI water and a variation of organic pollutant, oxidant, and dissolved NaHCO_3_ and NaCl, each in the concentration as described above. The reactions were carried out under constant shaking at 100 rpm and at a temperature of 30°C using a heated shaker plate (Titramax 1000 from Heidolph). The reactions were stopped after 18 h by removing the membrane from the solution, directly followed by analysis of the reaction solution (see Section S1.5).

S1.5 Analytical methods

We quantified the catalytic performance of the metal-decorated membranes through multiple parameters. For all reactions, the aromatic organic content of the reaction solution was determined via UV-Vis absorbance between ~250-310 nm (A_Aromatic_) using a Cary UV-Vis spectrophotometer from Varian. The aromatic removal (R_Aromatic_) was calculated as the relative decrease in A_Aromatic_ after 18 h reaction compared to A_Aromatic_ before the membrane was immersed in the reaction solution. Additionally, for all reactions the total organic carbon (TOC) content of the reaction solution was determined using a TOC-V CPN system from Shimadzu, which in-line acidified and degassed the samples. The TOC removal (R_TOC_) was calculated as the relative decrease in TOC after 18 h reaction compared to the TOC before the membrane was immersed in the reaction solution. For TC, we further determined the conversion degree by the relative decrease in its characteristic UV-Vis absorbance at 360 nm after 18 h reaction. For DF, we also quantified the formation of a colored degradation product via its absolute UV-Vis absorbance at 450 nm (A_450nm_, 1 cm light pathway).

For all experiments with PS (both with and without NaHCO_3_ and NaCl), we determined the amount of metal ions that leached from the respective metal-decorated membrane into the solution after 18 h reaction. For this, we used the photometric NANOCOLOR® test kits for Cr, Mn, Fe, Co, Ni, Cu and Zn from MACHEREY-NAGEL GmbH & Co. KG.

S2. Capillary flow porometry


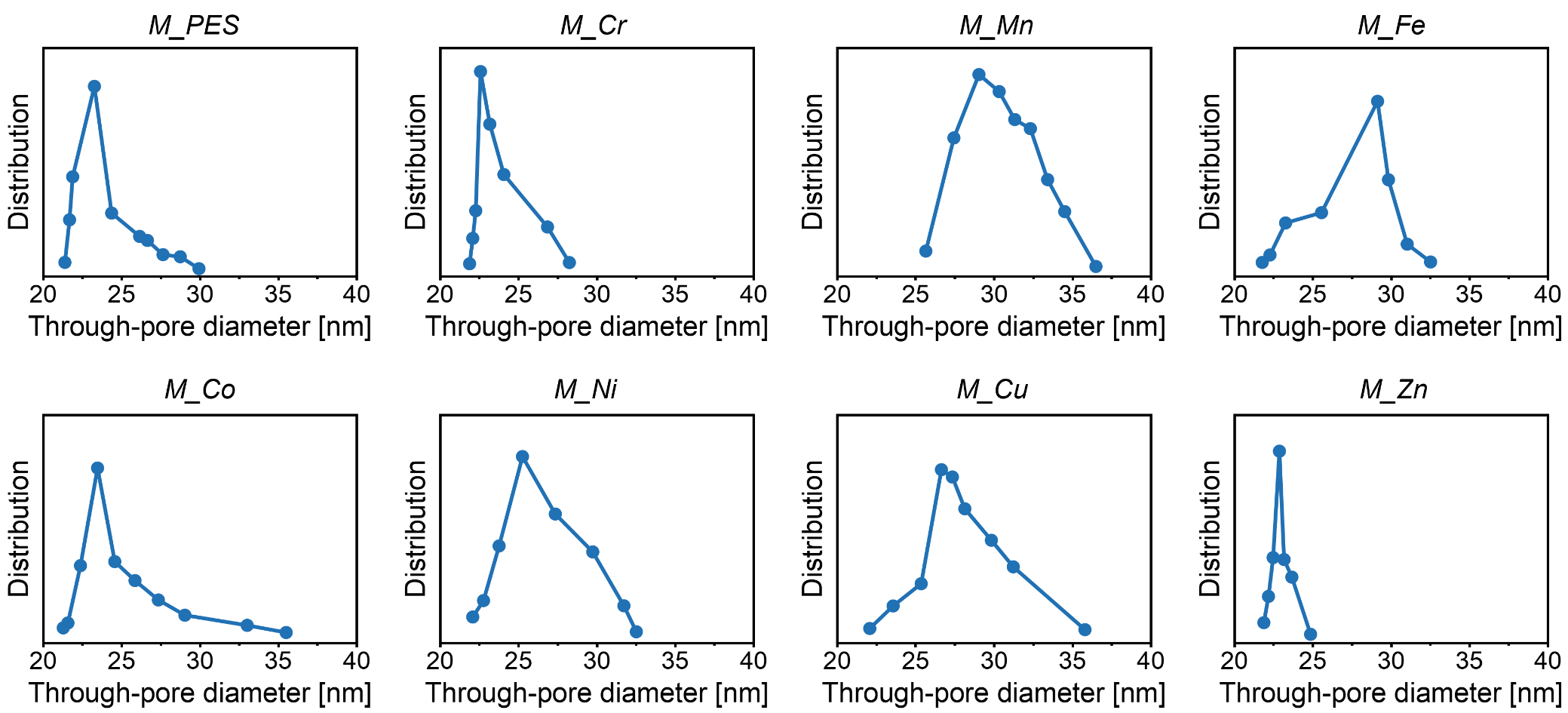
For the capillary flow porometry analysis, dry membrane samples were first wetted with Galwick (a short-chain polymerized hexafluoropropene). The wetted samples were then placed into a capillary flow porometer (model CFP-34RTG8A-X-6-LA, PMI). A liquid displacement pressure profile was obtained by measuring the volumetric gas flow at stepwise increasing nitrogen gas pressures for the wetted sample, followed by measurements at increasing nitrogen gas pressures for the fully dewetted sample. For each nitrogen pressure point that resulted in liquid displacement, the liquid transport-limiting diameter of the corresponding through-pores was calculated using Washburn’s equation (flow pore size). By comparing the measured flow volume associated with each through-pore diameter population to the theoretical volume of a pore at that diameter, the through-pore diameter distribution was determined. Both calculations were performed automatically by the PMI measurement software.

*Figure S1: Through-pore diameter distribution of the all-in-one prepared metal-decorated membranes as determined via capillary flow porometry.*

S3. XRD refinement and fitting

*Table S2: Lattice parameter and crystallite sizes (CS) calculated from the refined XRD patterns.*

|  | *M_Co (CoOOH)* | *M_Cu (CuO)* | *M_Zn (ZnO)* |
| --- | --- | --- | --- |
| a [Å] | 2.855 | 4.686 | 3.253 |
| b [Å] | - | 3.429 | - |
| c [Å] | 13.22 | 5.105 | 5.213 |
| β [°] | - | 99.04 | - |
| V [Å^3^] | 93.2 | 80.85 | 47.77 |
| CS [nm] | 15 | 14 | 23 |

S4. Mechanism of corrosion-based room-temperature oxidation


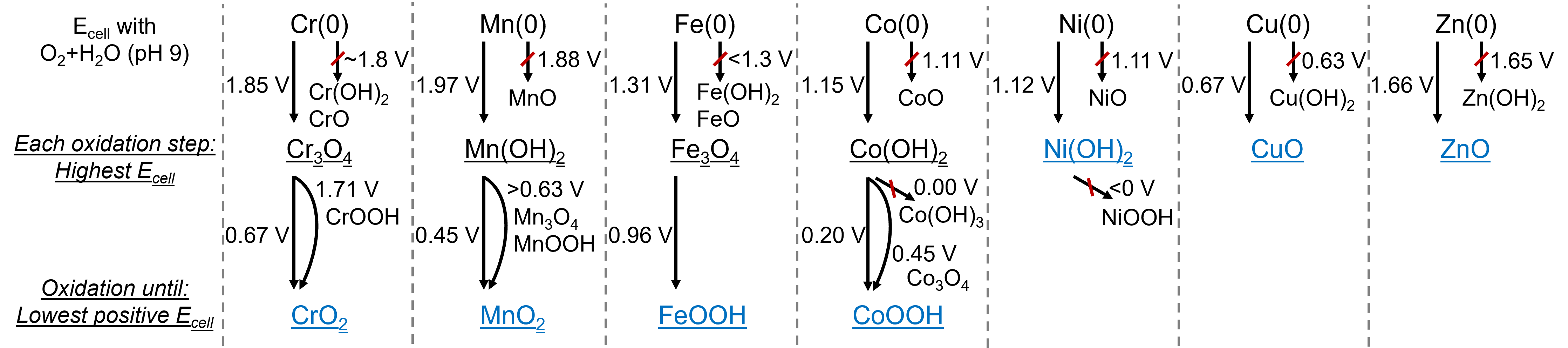
We calculated the theoretical cell potential (*E_cell_* at pH 9) for the formation of different metal oxidation products (known to occur in water in the presence of oxygen) using the Nernst equation and standard potentials (Figure S2) [1].

*Figure S2: Proposed mechanism of the corrosion-based room-temperature oxidation of membrane-immobilized M(0) in water at pH 9. The products that were experimentally identified are marked in blue. The standard potentials used for E_cell_ calculation have an uncertainty of around ± 0.005 V for Mn, Fe, Ni, Cu, Zn, and ± 0.05 V for Cr, Co* [1]*. The E_cell_ of M(III)_2_O_3_ species was not calculated because the hydrated M(III)OOH variants are known to form instead in water* [2]*.*

For Ni(0), Cu(0), and Zn(0), the *E_cell_* for the formation of the XRD-identified species Ni(OH)_2_, CuO and ZnO is higher than for NiO, Cu(OH)_2_ and Zn(OH)_2_, meaning that the energetically favored products were selectively formed (Figure S2). Additionally, nickel remained in its 2+ oxidation state, which matches the negative *E_cell_* value of -0.12 V for further oxidation of Ni(OH)_2_ to Ni(III)OOH under these conditions. However, for *M_Co* we observed the formation of CoOOH, even though the *E_cell_* for oxidation of Co(II)(OH)_2_ to Co(II,III)_3_O_4_ is higher than that for oxidation to Co(III)OOH. This suggests a stepwise oxidation process, where the most thermodynamically stable species forms at each step as the oxidation state increases (higher *E_cell_*). Then, oxidation of intermediately formed products continues (e.g., Co(II,III)_3_O_4_  to Co(III)OOH), until the potential of dissolved atmospheric oxygen can no longer drive any further reaction (lowest positive *E_cell_*). Notably, this stepwise process apparently did not result in particle passivation (e.g., stabilization of intermediate oxide species via lattice rearrangement), indicating that the immobilization of nanoparticles in the polymer matrix inhibits restructuring and conserves them in a state of high reactivity. Moreover, the exceptionally high metal utilization observed in the final membranes suggests that metal cations did not leach from the solid particles during the oxidation treatment, thereby excluding a cation dissolution-precipitation corrosion mechanism.

With this hypothesis regarding the oxidation mechanism, we can also explain the formation of Fe(III)OOH, instead of Fe(II,III)_3_O_4_, and the formation of MnO_2_ and CrO_2_ with +4 metal oxidation state, instead of +3 oxidation state products. It should also be noted that in a previous study of our own, we similarly fabricated a nickel-containing membrane [3]. In that study, we observed an optical band gap energy that suggested formation of NiO after treatment of the membrane at 50°C for 24 h in dry state, whereas in this study we dried samples at room temperature before characterization. This indicates that the dehydration of Ni(OH)_2_ to NiO can be achieved at higher temperatures, even in its immobilized state.

S5. Experimental data of the catalytic benchmarking

Several metrics for quantifying the organic pollutant oxidation process were evaluated (Figures S3-S6). We observed that assessing pollutant conversion alone, which cannot distinguish between single and multiple oxidation steps, can yield high apparent catalytic performances with minimal differences between the investigated metal oxides (as shown for TC in Figures S3-S6). On the other hand, the formation of degradation products can inversely correlate with the pollutant's degree of mineralization (as observed for DF in Figure S4), suggesting that a slower catalytic oxidation rate may lead to accumulation of intermediates formed early in the mineralization pathway. Additionally, total organic carbon (TOC) removal, which reflects mineralization, may underestimate the extent of oxidation for certain organic pollutants when stable degradation products are formed (as indicated for CFX in Figure S3). In contrast, the oxidation degree of the aromatic moieties provided a reliable metric for assessing the extent of catalytic pollutant degradation.

*
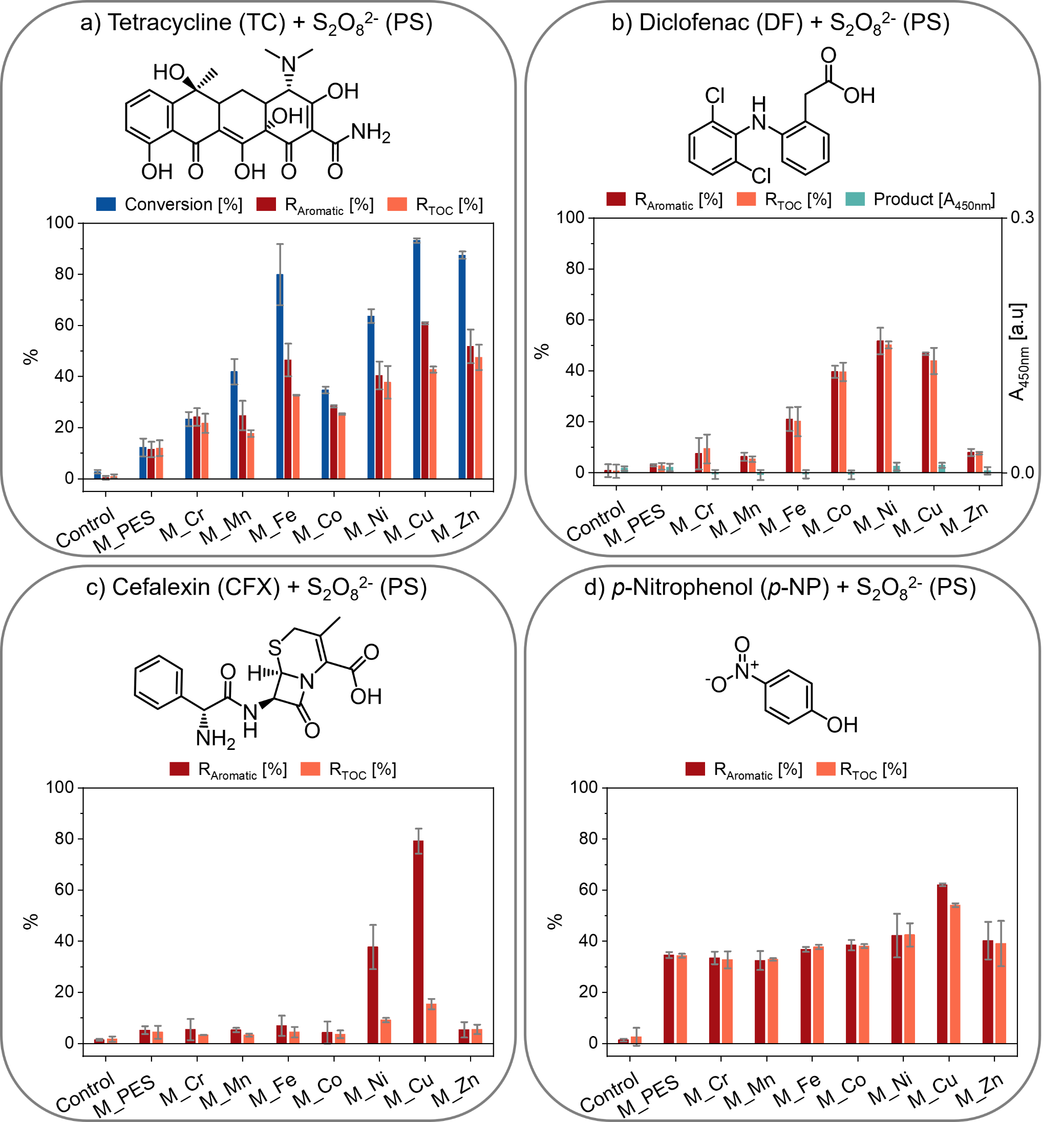
*

*Figure S3: Catalytic oxidation of different organic pollutants in water after 18 h at pH 7, using immersed membrane-immobilized transition metal oxides as catalyst and persulfate (PS) as oxidant. Average values and standard deviations were obtained by conducting each reaction separately with two samples of each membrane. a) Tetracycline (TC). b) Diclofenac (DF). c) Cefalexin (CFX). d) p-Nitrophenol (p-NP). Conditions: 18 h reaction time, 10 mL, 50 mg/L organic pollutant, 5 mM PS, pH 7, 3.14 cm^2^ membrane area, ~0.16 mg metal in each membrane.*

*
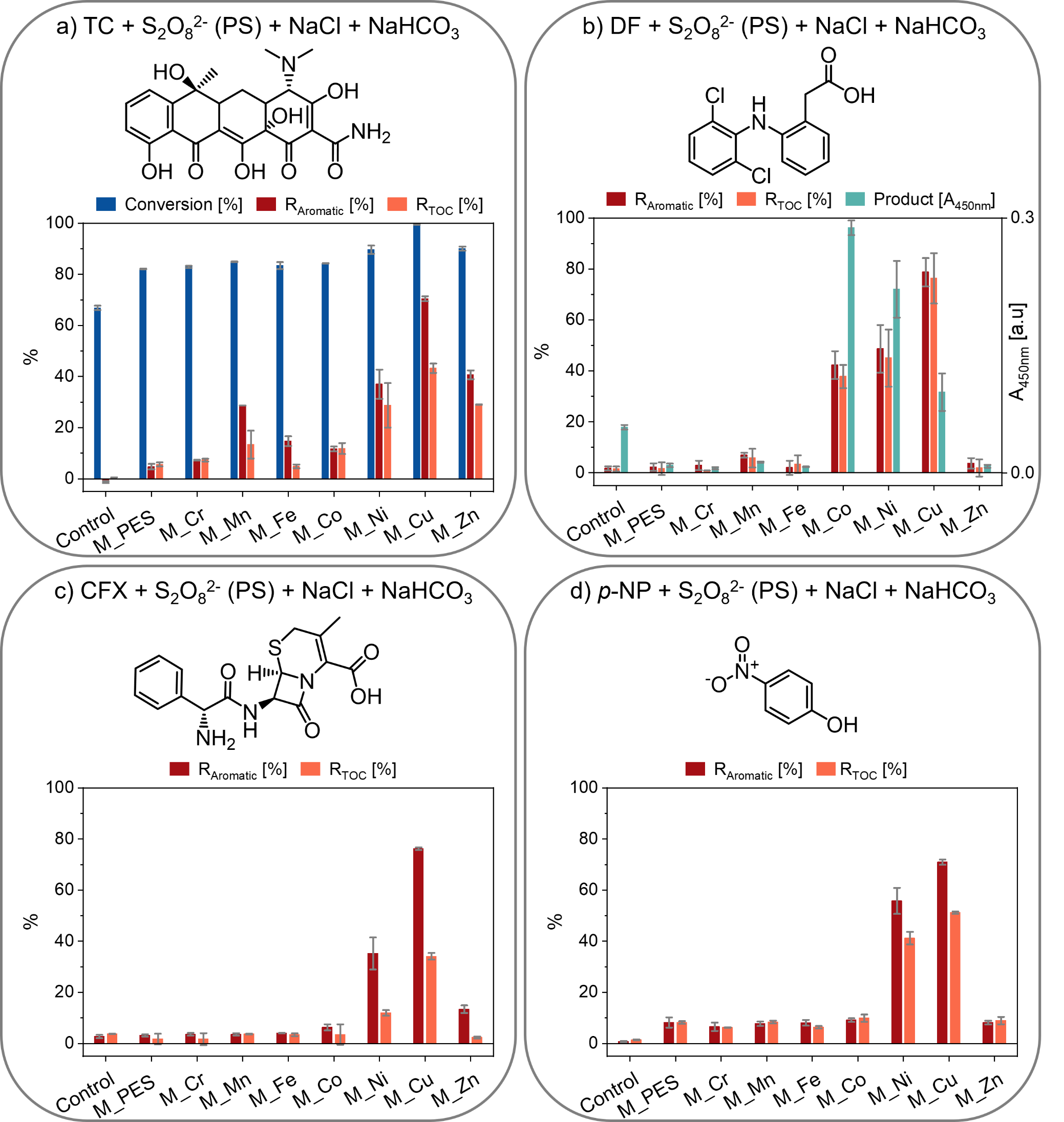
Figure S4: Catalytic oxidation of different organic pollutants in water after 18 h at pH 8 in presence of NaCl and NaHCO_3_, using immersed membrane-immobilized transition metal oxides as catalyst and PS as oxidant. Average values and standard deviations were obtained by conducting each reaction separately with two samples of each membrane. a) TC. b) DF. c) CFX. d) p-NP. Conditions: 18 h reaction time, 30°C, 10 mL, 50 mg/L organic pollutant, 5 mM PS, 5 mM NaHCO_3_, 3 mM NaCl, 3.14 cm^2^ membrane area, ~0.16 mg metal in each membrane.*


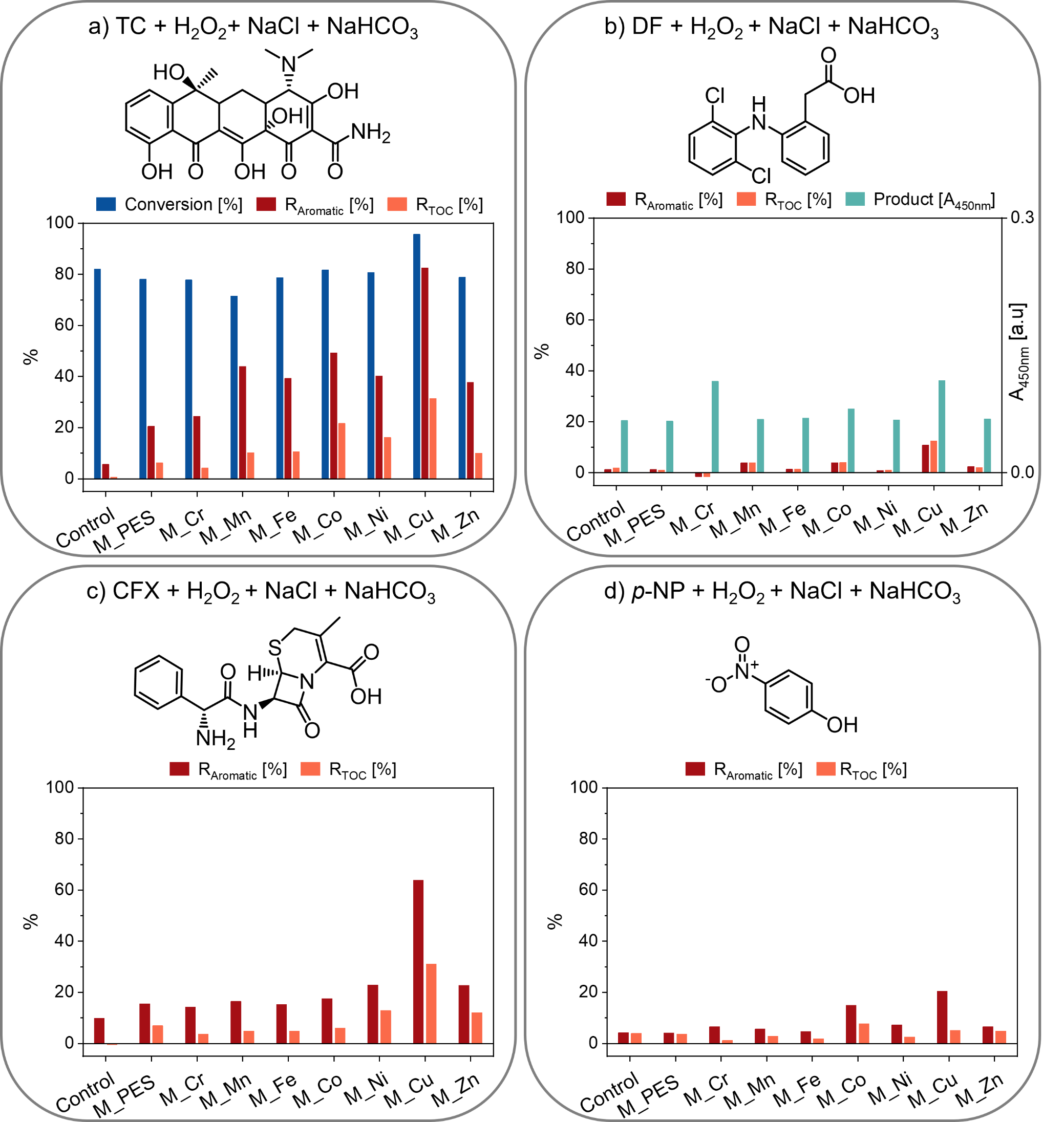
*Figure S5: Catalytic oxidation of different organic pollutants in water after 18 h at pH 8 in presence of NaCl and NaHCO_3_, using immersed membrane-immobilized transition metal oxides as catalyst and H_2_O_2_ as oxidant. a) TC. b) DF. c) CFX. d) p-NP. Conditions: 18 h reaction time, 30°C, 10 mL, 50 mg/L organic pollutant, 5 mM H_2_O_2_, 5 mM NaHCO_3_, 3 mM NaCl, 3.14 cm^2^ membrane area, ~0.16 mg metal in each membrane.*


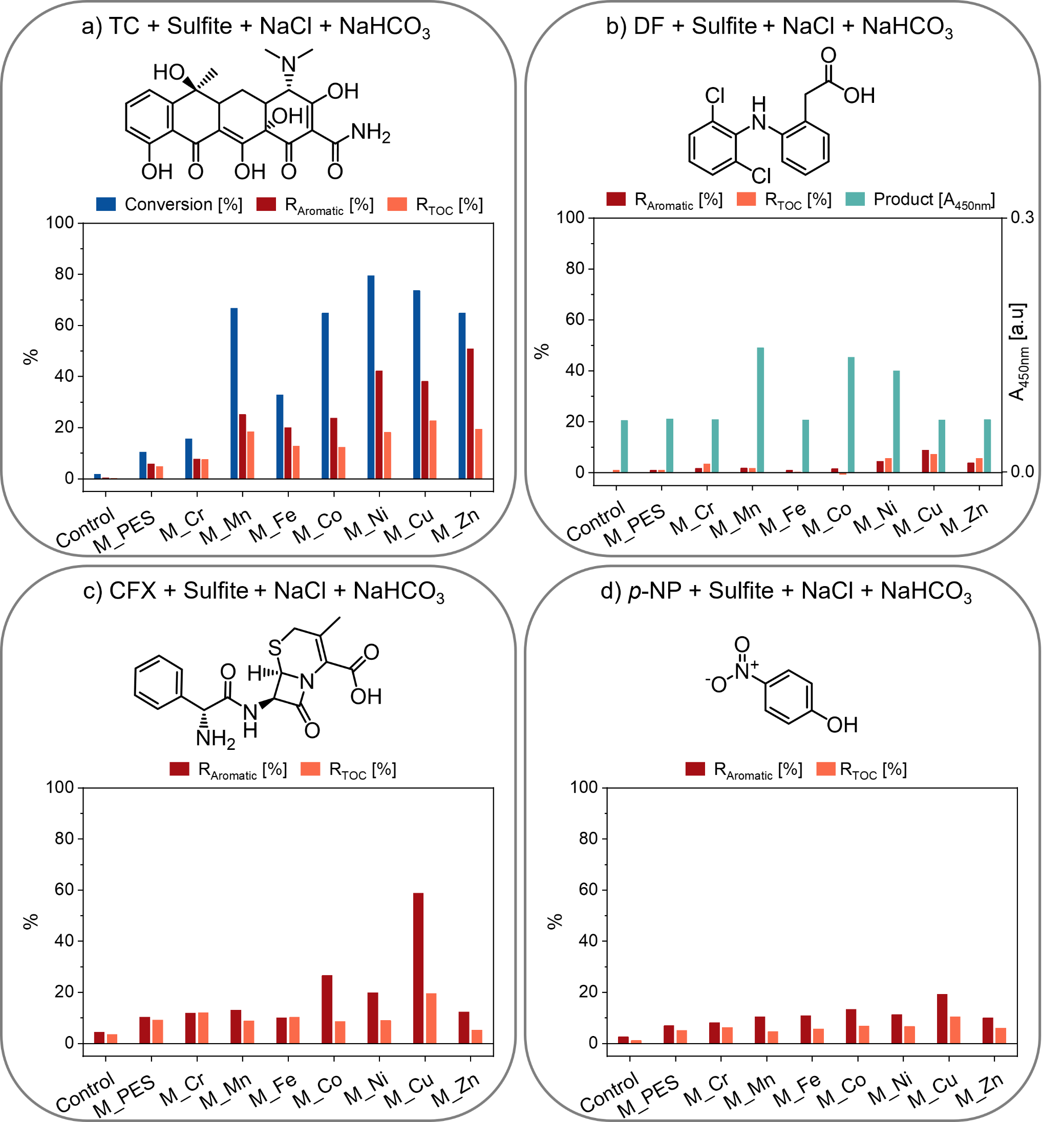
*Figure S6: Catalytic oxidation of different organic pollutants in water after 18 h at pH 8 in presence of NaCl and NaHCO_3_, using immersed membrane-immobilized transition metal oxides as catalyst and sulfite as oxidant. a) TC. b) DF. c) CFX. d) p-NP. Conditions: 18 h reaction time, 30°C, 10 mL, 50 mg/L organic pollutant, 5 mM sulfite, 5 mM NaHCO_3_, 3 mM NaCl, 3.14 cm^2^ membrane area, ~0.16 mg metal in each membrane.*


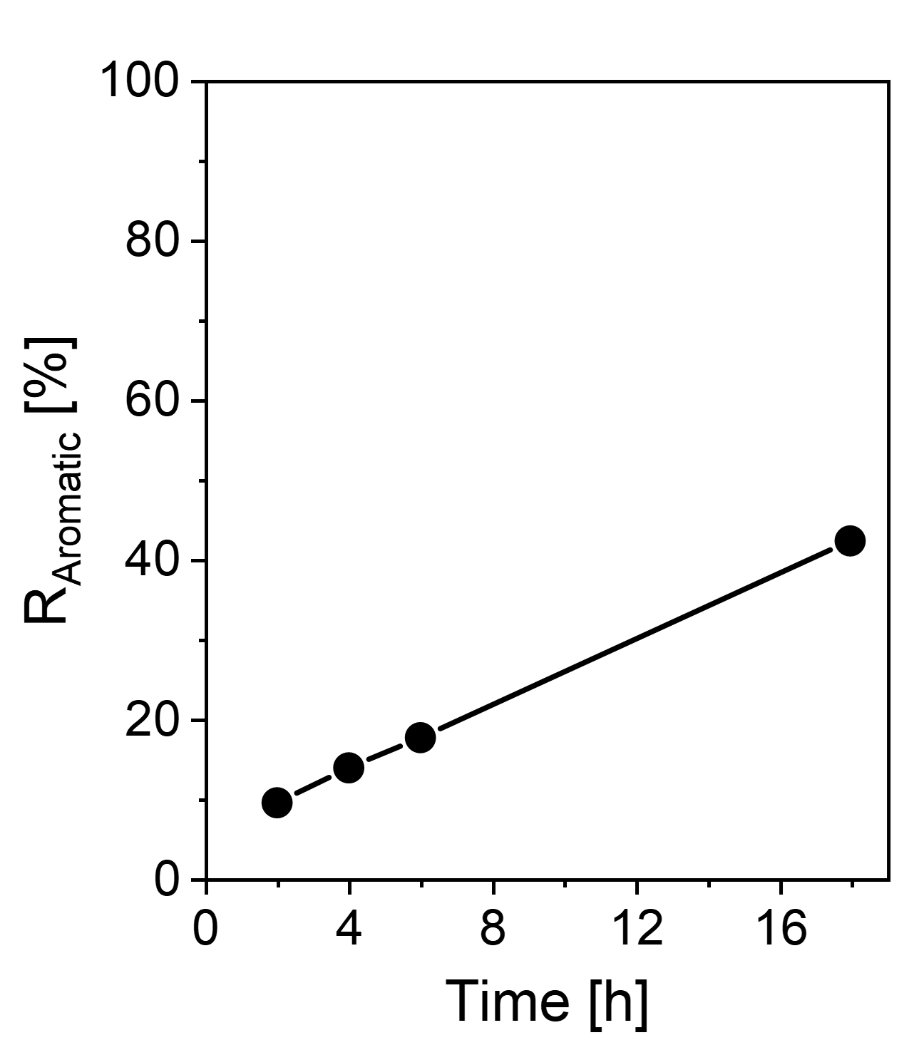
S6. Oxidation kinetic of DF with *M_Co*

*Figure S7: Catalytic aromatic oxidation degree of DF during 18 h at pH 8 in presence of NaCl and NaHCO_3_ with PS as oxidant, using M_Co as immersed catalyst. Conditions: 18 h reaction time, 10 mL, 50 mg/L DF, 5 mM PS, 5 mM NaHCO_3_, 3 mM NaCl, 3.14 cm^2^ membrane area, ~0.16 mg metal.*


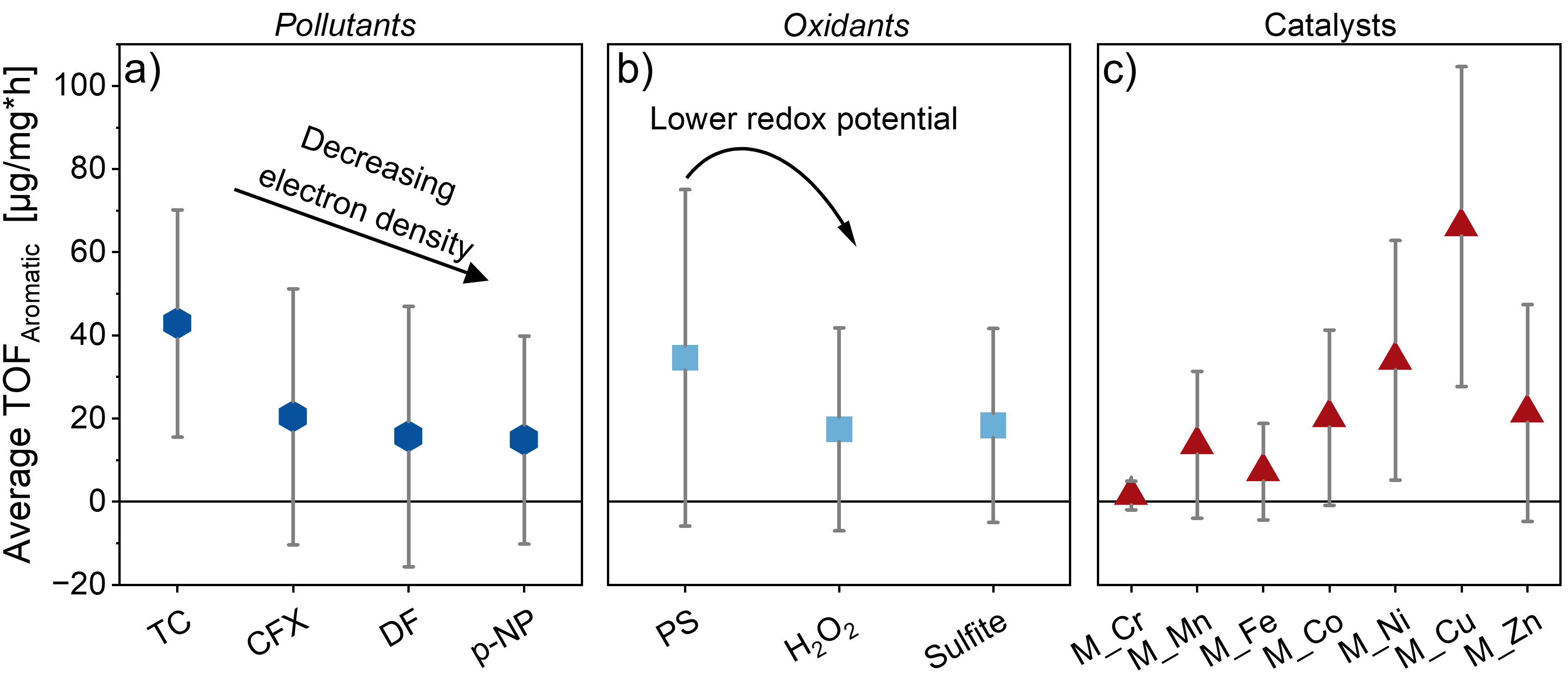
S7. Average TOF_Aromatic_ for individual parameters

*Figure S8: Average TOF_Aromatic_ for each individual parameter across all experiments of the benchmarking with NaCl and NaHCO_3_ at pH 8. a) Organic pollutants. b) Oxidants. c) Membrane-immobilized metal oxides. Conditions: 18 h reaction time, 10 mL, 50 mg/L organic pollutant, 5 mM oxidant, 5 mM NaHCO_3_, 3 mM NaCl, 3.14 cm^2^ membrane area, ~0.16 mg metal.*

S8. Modeling of catalytic oxidant activation activity

The Mulliken electronegativity (*Χ*) describes the relationship between ionization energy (IE) and electron affinity (EA) of an atom, molecule, or solid, and is defined as follows:

*Χ = 0.5 * (IE+EA) (S2)*

*Χ: Mulliken electronegativity [eV]; IE: ionization energy [eV]; EA: electron affinity [eV].*

In other words, *Χ* represents an energy level that lies midway between either valence and conduction band, or HOMO and LUMO. Matar et al. proposed an equation to calculate *Χ_M_* for transition metal cations [4], which they developed from an empirical relationship between *Χ_M_* and the charge number as well as ionic radius of a metal cation:

*Χ_M_ = (((0.274*z - 0.15*z*r - 0.01*r) + α) + 0.206) / 0.336 (S3)*

*Χ_M_: Mulliken electronegativity of metal cation [eV];* *z: charge number; r: ionic radius [Å]; α: element specific empirical constant.*

It should be noted that Equation S3 already includes conversion factors to express *Χ_m_* in eV.

In the framework of our model, we propose that individual metal active sites (MAS), which are surface-bound within the bulk metal oxide, activate oxidants through a catalytic redox cycle. In this cycle, MAS undergo one-electron transfer, switching between oxidation states and generating radicals. Therefore, we propose that MAS can be described as surface-bound metal cations, meaning their *Χ_M_* values can be calculated using Equation S3. Under this premise, the *r* stated in Equation S3 refers to the ionic radius of a metal cation with charge *z* when in a coordination state that is equal to that of metal sites in the crystal structure of the respective metal oxide.

We further assumed that the investigated metal oxides can be characterized as semiconductors with a uniform band gap, where the localized energy states at individual surface-bound MAS still lie in that band gap. This assumption is made because the gap between valence and conduction bands can primarily be attributed to the influence of lattice oxygen and the lattice structure, which we propose remain unchanged when a MAS cycles between oxidation states during oxidant activation. The lattice oxygen-metal interaction in metal oxides is usually relatively robust and does not instantaneously change during redox processes, only after phase transitions or oxygen migration occur.

Consequently, the *IE_MAS_*, defined as the energy required to eject an electron from an individual MAS, can be calculated as follows:

*IE_MAS_ = Χ_M_ + 0.5 * E_g_ (S4)*

*IE_MAS_: ionization energy of metal active site [eV]; Χ_M_: Mulliken electronegativity of metal cation [eV]; E_g_: observed optical band gap energy of the bulk metal oxide [eV] (cf. Figure 3 in the manuscript).*

It should be noted that for all membrane-immobilized metal oxides the measured optical band gap energy was employed with the exception of CrO_2_, for which we used *E_g_* from literature [4].

Furthermore, we propose that activation of S_2_O_8_^2-^ (PS) and H_2_O_2_ involves a M(X) → M(X+1) → M(X) transition of MAS, while SO_3_^2-^ (sulfite) activation involves a M(X) → M(X-1) → M(X) transition due to its negative redox potential. Additionally, we propose that electron ejection from MAS in their M(X) state, respectively M(X-1) state is the rate-determining step of oxidant activation, and that oxidant activation is the rate-determining step for organic pollutant degradation.

Based on this, we developed a modified Arrhenius equation, connecting the oxidant-specific average *TOF_Aromatic_* of a metal oxide to its oxidant-specific *IE_MAS_*:

*TOF_Aromatic_ = A * e^((pf * -IE_MAS_) / k_b_ * T ) (S5)*

*TOF_Aromatic_: oxidant-specific average TOF_Aromatic_ of the metal oxide [µg/mg*h]; A: maximum catalytic TOF_Aromatic_ achievable with the employed oxidant under the investigated reaction conditions [µg/mg*h]; pf: proportionality factor, estimated as 0.1, which converts IE values to the order of magnitude commonly observed for activation energies; IE: oxidant-specific IE_MAS_ of the employed metal oxide [eV]; k_b_: Boltzmann constant [eV/K]; T: Reaction temperature [K].*

In Equation S5, we incorporated a proportionality factor, *pf*, which we set to 0.1 as an estimate. Activation energies commonly reported for catalytic reactions are in the range of 30-100 kJ/mol, which corresponds to 0.31-1.03 eV. In contrast, IE values commonly reported for bulk metal oxides are in the range of 5-10 eV [5], as are the *IE_MAS_* values that Equations S3 and S4 predict. Due to the observation that the difference between common activation energies and IE values is exactly an order of magnitude, we introduced *pf* with 0.1 in the modified Arrhenius equation.

To model *IE_MAS_* via Equation S5 from experimentally determined *TOF_Aromatic_* values of different metal oxides, the band gap energy of the metal oxides is needed (cf. Equation S4), as well as the charge number and ionic radius of the metal cation that constitutes the MAS during the rate-determining reaction step (cf. Equation S3). As stated above, during oxidant activation involving a catalytic redox cycle, we propose that electron release is the rate-determining step for the redox cycling of MAS. The oxidation state, reflecting the charge number, of MAS during electron ejection is either M(X) (for PS and H_2_O_2_), identical to the metal oxidation state in the bulk oxide, or M(X-1) (for sulfite), one electron less than the initial oxidation state. The ionic radius, on the other hand, depends on the coordination environment of MAS during electron ejection, and, consequently on the reaction conditions. The ground-state coordination number (CN) of MAS is determined by the CN of metal sites in the bulk metal oxide, corresponding to the number of coordinating lattice oxygen. However, we assumed that this CN is reduced by 1 for MAS, because they are surface-bound, meaning they are not coordinated by lattice oxygen atoms on all sides.

For an individual MAS to eject an electron during redox cycling, it needs to from a complex with one or more molecules that act as electron acceptors. Both the oxidants S_2_O_8_^2-^ and H_2_O_2_ act themselves as electron acceptors. In contrast, during SO_3_^2-^ activation, SO_3_^2-^ itself acts as the reaction partner only during the M(X) → M(X-1) transition, while dissolved O_2_ likely acts as the electron-accepting reaction partner for the rate-determining M(X-1) → M(X) transition. Overall, we propose that the effective ionic radius of MAS during electron ejection differs from their ground-state ionic radius, resulting from an increase in CN due to complexation by electron-accepting molecules. For example, the CN of Cu^2+^ in CuO is 4, meaning that when surface-bound Cu^2+^ acts as MAS, the ground-state CN is 3. For the hypothetical case that the CN of Cu^2+^ increases by 3 during electron ejection due to complexation with electron-accepting molecules, its ionic radius should be equal to the ionic radius of Cu^2+^ bound in a bulk crystal structure with CN 6 [6].

In conclusion, for the modeling of *IE_MAS_* via Equation S4 we employed the following conditions: As modeling variable to obtain *IE_MAS_*, we used *Χ_M_*, and as modeling objective to be solved we used an as low as possible residual sum of squares of observed *TOF_Aromatic_* values compared to *TOF_Aromatic_* values calculated using Equation S4. *Χ_M_* was calculated using Equation S3 with a static charge number, corresponding to either the M(X) state for S_2_O_8_^2-^ and H_2_O_2_ as oxidants, or the M(X-1) state for SO_3_^2-^. As underlying variable of *Χ_M_*, we used the ionic radius of MAS, with upper and lower boundaries corresponding to the ionic radius that the respective metal cation would have in a crystal structure with CN ranging from -1 to +2 compared to the CN in the bulk metal oxide (Table S3-S5) [6]. Here, -1 represents a surface-accessible MAS with no additional surface coordination (ground-state CN), while +2 represents MAS that are surface-coordinated by three different functional groups or molecules. We set the upper CN boundary to +2, ensuring the CN relative to the bulk metal oxide is increased from 4 or 6 only to 6 or 8, respectively, which are typically favored and stable CN states. As additional modeling variable, we further employed parameter *A* as stated in Equation S4.

Modeling was then conducted using the “evolutionary” method of the Excel Solver function with 0.00000001 “precision” (a lower number corresponds to a higher precision), 0.000001 “convergence”, 0.1 “mutation rate”, 500 “population size” and with “random seed” set to 0. It should be noted that as variables, *A* as well as the *IE_MAS_* of all metal oxides were modeled at the same time. In the “evolutionary” method of the Excel Solver this is achieved by stepwise optimization of a set of populations, each containing different input values for all variables.

In Table S3-S5, all employed parameters and the values obtained from the modeling are shown. Furthermore, Table S6 presents the fit parameters for Equation S5 that resulted from the modeling. The pre-exponential factor *A* in Equation S5 corresponds to the highest catalytic *TOF_Aromatic_* achievable with the respective oxidant under the investigated reaction conditions. In other words, this represents the hypothetical diffusion-limited *TOF_Aromatic_* a catalyst with zero activation energy would exhibit. Converted into mol per mol per s, the *A* values derived from our modeling fall within the range of 10^4^-10^6^ s^-1^ (Table S6). Notably, this range is consistent with the TOFs reported for the fastest known heterogeneous enzyme reactions (10^4^-10^6^ s^-1^) [7], which are considered to be at or near the diffusion limit. This agreement supports the physical realism of our modeling results.

*Table S3: Modeled ionic radii, CN, Χ_M_ and IE_MAS_ for M(X) active sites in contact to S_2_O_8_^2-^ as oxidant and parameters used for modeling.*

| Catalyst | Static parameter: a | Static parameter: M(X) charge number | M(X) CN bulk | *Variable parameter:*  *M(X) lower boundary* ionic radius [Å] (corresponding CN) | *Variable parameter:*  *M(X) upper boundary* ionic radius [Å] (corresponding CN) | Static parameter: 0.5 * E_g_ (eV) | Modeled: M(X) ionic radius [Å] | Modeled: CN | Modeled: M(X) X_M_ [eV] | Modeled: M(X) IE_MAS_ [eV] |
| --- | --- | --- | --- | --- | --- | --- | --- | --- | --- | --- |
| CrO_2_ | 0.2 | +4 | 6 | 0.48 (5) | 0.69 (8) | 1.50 | 0.53 | 5.70 | 6.49 | 7.99 |
| MnO_2_ | 0.095 | +4 | 6 | 0.46 (5) | 0.67 (8) | 1.10 | 0.54 | 6.13 | 6.16 | 7.25 |
| FeOOH | 0.276 | +3 | 6 | 0.48 (5) | 0.69 (8) | 1.27 | 0.48 | 5.00 | 6.20 | 7.47 |
| CoOOH | 0.233 | +3 | 6 | 0.475 (5) | 0.685 (8) | 1.16 | 0.55 | 6.02 | 5.98 | 7.14 |
| Ni(OH)_2_ | 0.168 | +2 | 6 | 0.62 (5) | 0.83 (8) | 1.91 | 0.83 | 8.00 | 4.96 | 6.86 |
| CuO | 0.408 | +2 | 4 | 0.50 (3) | 0.71 (6) | 0.93 | 0.68 | 5.52 | 5.81 | 6.74 |
| ZnO | 0.263 | +2 | 4 | 0.53 (3) | 0.74 (6) | 1.65 | 0.59 | 3.86 | 5.46 | 7.11 |

| Catalyst | Static parameter: a | Static parameter: M(X) charge number | M(X) CN bulk | *Variable parameter:*  *M(X) lower boundary* ionic radius [Å] (corresponding CN) | *Variable parameter:*  *M(X) upper boundary* ionic radius [Å] (corresponding CN) | Static parameter: 0.5 * E_g_ (eV) | Modeled: M(X) ionic radius [Å] | Modeled: CN | Modeled: M(X) X_M_ [eV] | Modeled: M(X) IE_MAS_ [eV] |
| --- | --- | --- | --- | --- | --- | --- | --- | --- | --- | --- |
| CrO_2_ | 0.2 | +4 | 6 | 0.48 (5) | 0.69 (8) | 1.50 | 0.68 | 7.88 | 6.21 | 7.71 |
| MnO_2_ | 0.095 | +4 | 6 | 0.46 (5) | 0.67 (8) | 1.10 | 0.65 | 7.72 | 5.95 | 7.05 |
| FeOOH | 0.276 | +3 | 6 | 0.48 (5) | 0.69 (8) | 1.27 | 0.69 | 8.00 | 5.91 | 7.18 |
| CoOOH | 0.233 | +3 | 6 | 0.475 (5) | 0.685 (8) | 1.16 | 0.68 | 7.92 | 5.80 | 6.96 |
| Ni(OH)_2_ | 0.168 | +2 | 6 | 0.62 (5) | 0.83 (8) | 1.91 | 0.62 | 5.00 | 5.15 | 7.06 |
| CuO | 0.408 | +2 | 4 | 0.50 (3) | 0.71 (6) | 0.93 | 0.71 | 6.00 | 5.78 | 6.71 |
| ZnO | 0.263 | +2 | 4 | 0.53 (3) | 0.74 (6) | 1.65 | 0.63 | 4.50 | 5.41 | 7.05 |

*Table S4: Modeled ionic radii, CN, Χ_M_ and IE_MAS_ for M(X) active sites in contact to H_2_O_2_ as oxidant and parameters used for modeling.*

*Table S5: Modeled ionic radii, CN, Χ_M_ and IE_MAS_ for M(X-1) active sites in contact to O_2_ as electron acceptor during SO_3_^2-^ activation and parameters used for modeling.*

| Catalyst | Static parameter: a | Static parameter: M(X-1) charge number | M(X) CN bulk | *Variable parameter:*  *M(X-1) lower boundary* ionic radius [Å] (corresponding CN) | *Variable parameter:*  *M(X-1) upper boundary* ionic radius [Å] (corresponding CN) | Static parameter: 0.5 * E_g_ (eV) | Modeled: M(X-1) ionic radius [Å] | Modeled: CN | Modeled: M(X-1) X_M_ [eV] | Modeled: M(X-1) IE_MAS_ [eV] |
| --- | --- | --- | --- | --- | --- | --- | --- | --- | --- | --- |
| CrO_2_ | 0.2 | +3 | 6 | 0.545 (5) | 0.755 (8) | 1.50 | 0.76 | 8.00 | 5.60 | 7.10 |
| MnO_2_ | 0.095 | +3 | 6 | 0.51 (5) | 0.72 (8) | 1.10 | 0.61 | 6.38 | 5.49 | 6.58 |
| FeOOH | 0.276 | +2 | 6 | 0.54 (5) | 0.75 (8) | 1.27 | 0.66 | 6.66 | 5.44 | 6.71 |
| CoOOH | 0.233 | +2 | 6 | 0.58 (5) | 0.79 (8) | 1.16 | 0.63 | 5.70 | 5.34 | 6.50 |
| Ni(OH)_2_ | 0.168 | +1 | 6 | 0.66 (5) | 0.87 (8) | 1.91 | 0.75 | 6.26 | 4.55 | 6.46 |
| CuO | 0.408 | +1 | 4 | 0.53 (3) | 0.74 (6) | 0.93 | 0.53 | 3.00 | 5.37 | 6.29 |
| ZnO | 0.263 | +1 | 4 | 0.79 (3) | 1.00 (6) | 1.65 | 0.88 | 4.29 | 4.77 | 6.42 |

*Table S6: Fit parameters for Equation S4 that resulted from modeling IE_MAS_ to the observed average TOF_Aromatic_ of the different membrane-immobilized metal oxides with each of the employed oxidants. To calculate A in s^-1^, a generic molecular weight of 300 g/mol was assumed for the pollutant and 60 g/mol for the catalyst.*

| Oxidant | *A* [µg/mg*h] | *A* [s^-1^] | *pf* | Total sum of squares | Residual sum of squares |
| --- | --- | --- | --- | --- | --- |
| S_2_O_8_^2-^ | 2*10^13^ | 1*10^6^ | 0.1 | 17340 | 3 |
| H_2_O_2_ | 7*10^12^ | 4*10^5^ | 0.1 | 3747 | 1 |
| SO_3_^2-^ | 1*10^12^ | 6*10^4^ | 0.1 | 3218 | 3 |

S9. Metal active site surface coordination

During the M(X) → M(X+1) transition with S_2_O_8_^2-^ and H_2_O_2_ as oxidants, both these oxidants itself act as electron acceptor and need to be coordinated to the surface for this transitions to occur. In contrast, during the M(X-1) → M(X) transition during activation of SO_3_^2-^ as an oxidant, SO_3_^2-^ does not act as the electron acceptor, and this role is likely fulfilled by dissolved O_2_. Generally, surface-accessible MAS have a lower CN than the metal sites in the bulk metal oxide, typically resulting in a CN that is reduced by 1. Based on this, we calculated the effective surface coordination of MAS during the electron ejection step from the modeled CN of MAS as follows (cf. Table S3-S5):

*Surface coordination = CN_MAS_ - (CN_Bulk_ - 1) (S6)*

*Surface coordination: effective surface coordination of M(X) or M(X-1) during the electron ejection step of the catalytic redox cycle; CN_MAS_: modeled CN of M(X) or M(X-1); CN_Bulk_: CN of metal sites in the bulk metal oxide.*

S10. Impact of input parameter variations on modeling results

To analyze if *TOF_Aromatic_* variations result in prediction of similar activity trends among the different membrane-immobilized metal oxides, we employed the upper and lower standard deviation boundaries of the oxidant-specific *TOF_Aromatic_* values as input for our modeling procedure (cf. Sections S8 and S9). Figures S9–S11 show the results of this analysis for the three different oxidants. The most obvious issues arise from the lower deviation of the *TOF_Aromatic_*, which can reach negative values and/or reduce the differences between the metal oxides. This results in a poor fit between experimental *TOF_Aromatic_* values and these obtained from modeling (cf. Equation S5), and, consequently, unreliable *IE_MAS_* and surface *CN_MAS_* predictions. Nevertheless, for PS (Figure S9) and H_2_O_2_ (Figure S10), the *CN_MAS_* obtained from the lower deviation of *TOF_Aromatic_* values exhibit trends similar to those obtained from the average *TOF_Aromatic_* values. For the upper deviation of *TOF_Aromatic_*, the modeling produces oxidant-specific *IE_MAS_* values that are consistent with the modified Arrhenius equation (cf. Equation S5) and comparable to those obtained from the average *TOF_Aromatic_*. Moreover, for all oxidants the *CN_MAS_* predicted from the average and upper deviation *TOF_Aromatic_* values depict the same trends. For PS and sulfite (Figure S11), however, most of the surface *CN_MAS_* values derived from the upper deviation are extremely high, nearing or reaching the limit of 3 (this corresponds to an overall CN of 6 or 8, which is usually an energetically favored state and was set as limit to prevent overfitting). This suggests that the highest *TOF_Aromatic_* values observed for each metal oxide correspond to scenarios where MAS are coordinated by more molecules than anticipated in our framework. A possible explanation is that these cases involve specific catalyst-pollutant interactions, which contribute to the coordination environment alongside the dominant catalyst-oxidant interactions. Additionally, it is important to emphasize that our modeling adopts a relative approach, meaning the predicted values should not be interpreted as absolute physical parameters but rather as indicators for material property trends among the different metal oxides under the investigated conditions.

The relative approach was adopted due to the pre-exponential factor *A* of the modified Arrhenius equation (cf. Equation S5). *A* should theoretically be an intrinsic system constant when we define it as the maximum (diffusion-limited) catalytic *TOF_Aromatic_* achievable under the employed reaction conditions. However, *A* was modeled alongside the *IE_MAS_* values, making it sensitive to variations in *TOF_Aromatic_* inputs and, thus, introducing an additional error. Therefore, to strengthen predictions from our framework, methods to accurately calculate *A* values need to be developed (e.g., deriving *A* from collision frequency calculations), and *A* should then be treated as a constant during modeling.


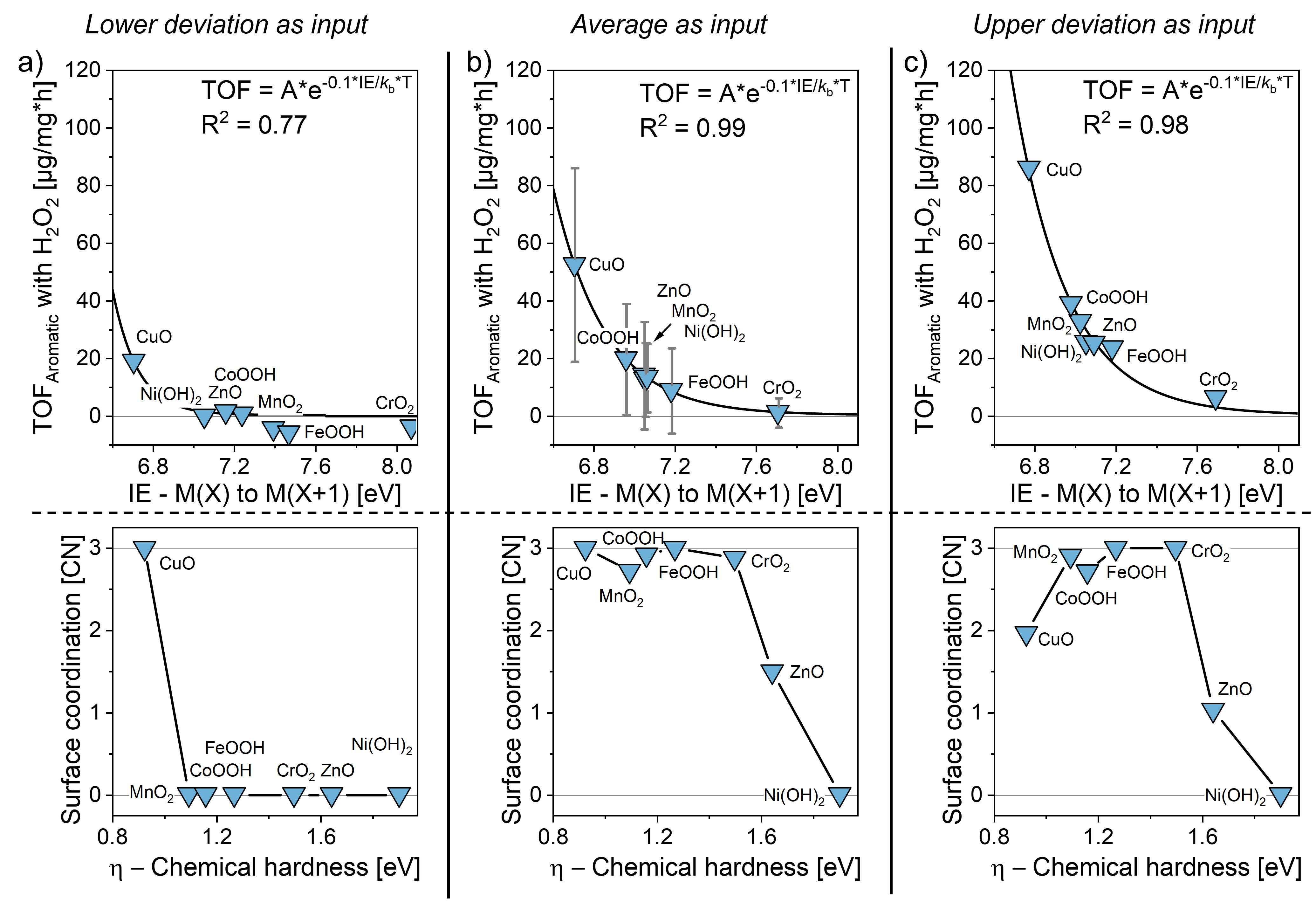

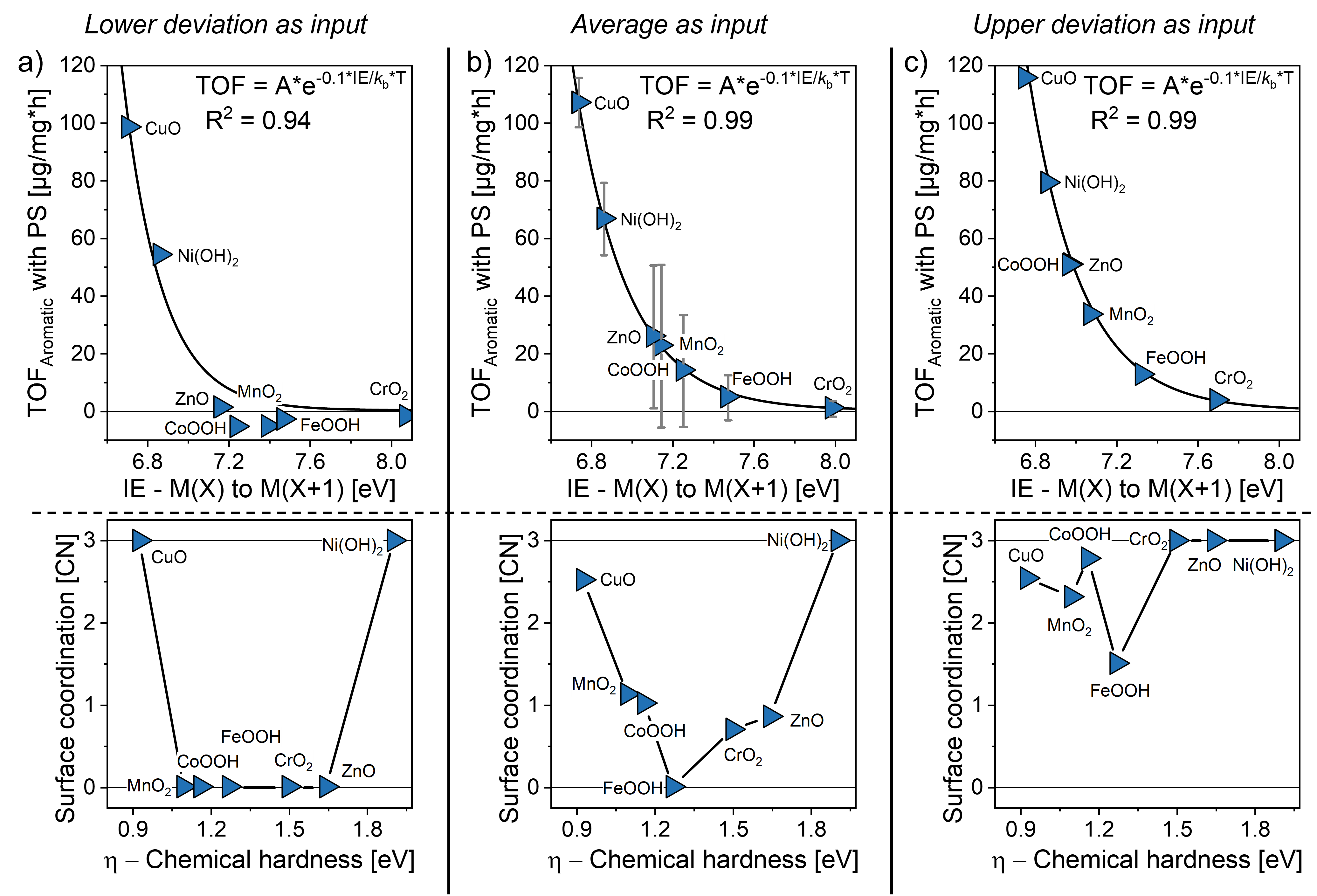
*Figure S9: Modeled IE_MAS_ and CN_MAS_ of membrane-immobilized metal oxides with PS as oxidant. a) Using the lower standard deviation boundary of TOF_Aromatic_ values as input. b) Using the average TOF_Aromatic_ values as input. c) Using the upper standard deviation boundary of TOF_Aromatic_ values as input.*

*Figure S10: Modeled IE_MAS_ and CN_MAS_ of membrane-immobilized metal oxides with H_2_O_2_ as oxidant. a) Using the lower standard deviation boundary of TOF_Aromatic_ values as input. b) Using the average TOF_Aromatic_ values as input.* *c) Using the upper standard deviation boundary of TOF_Aromatic_ values as input.*

*
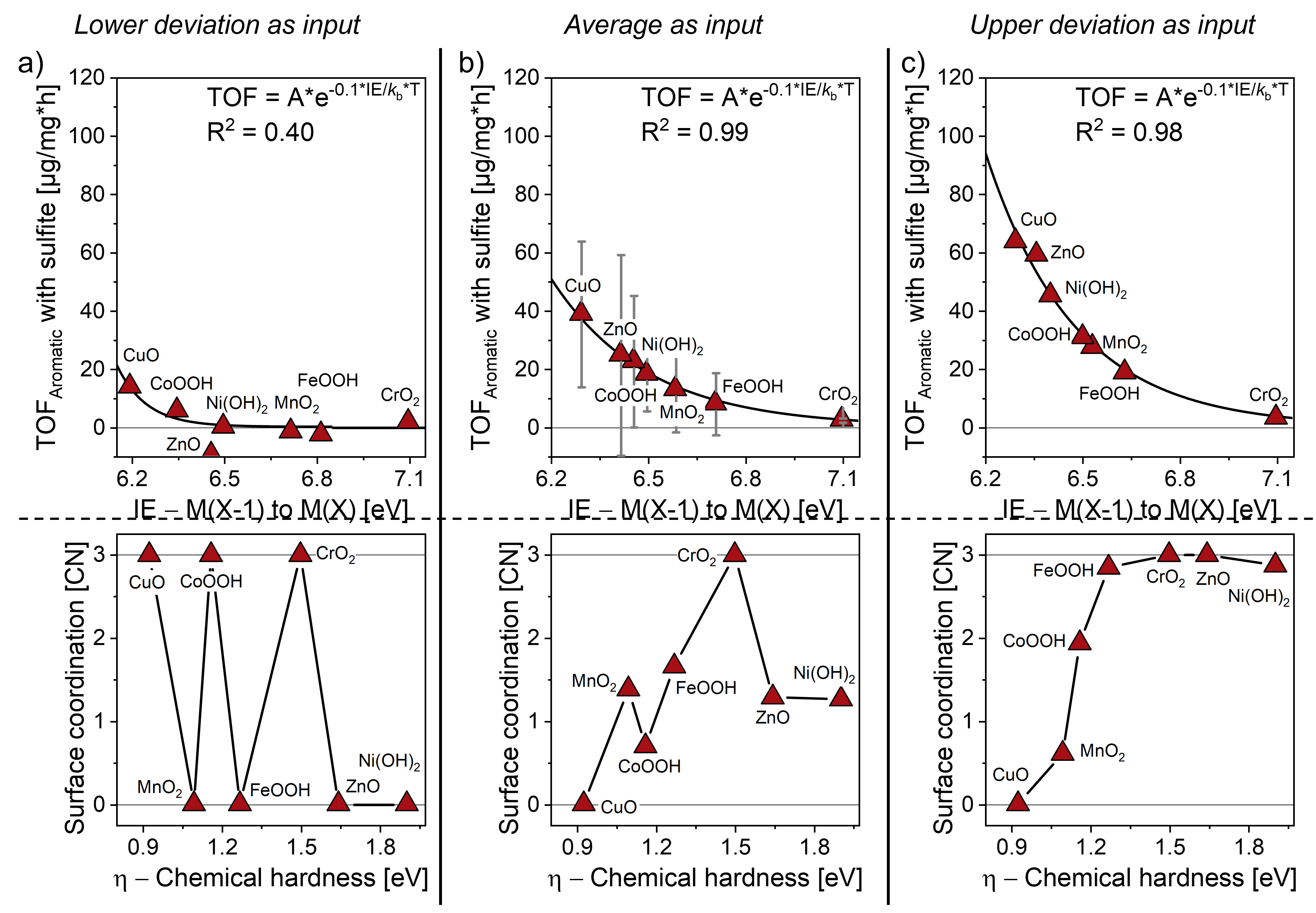
Figure S11: Modeled IE_MAS_ and CN_MAS_ of membrane-immobilized metal oxides with sulfite as oxidant. a) Using the lower standard deviation boundary of TOF_Aromatic_ values as input. b) Using the average TOF_Aromatic_ values as input.* *c) Using the upper standard deviation boundary of TOF_Aromatic_ values as input.*

S11. Validating activity prediction with the framework

The framework was developed using data from key transition metal oxides, all tested under specific reaction conditions. Therefore, we sought to further validate the framework's ability to predict the catalytic activity of different catalysts under varied conditions. The Mulliken electronegativity of M(II) sites increases in the following order: Mn < Ni < Cr < Zn < Co < Fe < Cu. Our framework predicts that in cases of an identical electronic environment in the bulk oxide (identical band gap energy), a lower Mulliken electronegativity of MAS directly correlates with a lower *IE_MAS_* (cf. Equation S4) and thus higher oxidant activation activity (cf. Equation S5). Consequently, doping M(II) active sites into a bulk oxide such as Fe(III)OOH should yield catalysts with the following activity trend: Mn > Ni > Cr > Zn > Co > Cu. Both Fe_2_O_3_ and FeOOH are known to form solid solutions by incorporating secondary metals in the +2 oxidation state. In this substitution in FeOOH, charge neutrality is maintained, and Fe(III)-O lattice positions are replaced by M(II)-OH [8–10]. However, we excluded Cr from this doping strategy, as it is known to form passivation layers during oxidation of Cr-Fe alloys.

We synthesized a set of membrane-immobilized metal-doped FeOOH species using the *all-in-one* method (same conditions as described in Section S1.2). We also added 2 wt% of polyvinylpyrrolidine (PVP) to the casting solution, which is a common additive employed as both membrane porogen and capping agent for metal particles. The aim was to validate our framework using catalysts with different secondary properties than those in the main text, such as altered support porosity and PVP-capping. Our framework describes catalytic activity of metal oxides based on their intrinsic compositional and structural properties. Therefore, while addition of PVP is likely to alter the absolute activity of the metal oxides (e.g. due to altering particle size or accessible surface area), the framework should still be able to predict the activity trend across a series of catalysts all prepared with PVP. To generate metal-doped FeOOH, we simply combined the iron precursor with a secondary metal precursor in the casting solution, followed by *in situ* reduction to metallic alloy particles, membrane preparation, and post-treatment oxidation. To ensure that the base properties of FeOOH are retained, we doped the iron particles with only 10 mol% of the secondary metal (49.5 µmol iron and 5.5 µmol secondary metal per 1 g casting solution, cf. Table S1). In Table S7 the base properties (determined as described in Section S1) of the as-synthesized membrane-immobilized catalysts are shown.

*Table S7: Determined thickness, porosity, and metal loading for the metal-doped FeOOH-decorated membranes. Thickness and porosity were determined for two samples of each membrane and the average and standard deviations are shown.*

| Membrane | Thickness [µm] | Porosity [%] | Fe loading [µg/cm^2^] | Secondary metal loading [µg/cm^2^] |
| --- | --- | --- | --- | --- |
| *S_FeOOH* | 130 ± 2.1 | 84 ± 2 | 52 |  |
| *S_Mn_FeOOH_* | 133 ± 2.1 | 96 ± 2 | 47 | 2.1 |
| *S_Co_FeOOH_* | 142 ± 2.1 | 92 ± 1 | 54 | 3.6 |
| *S_Ni_FeOOH_* | 142 ± 2.1 | 87 ± 1 | 55 | 3.0 |
| *S_Cu_FeOOH_* | 132 ± 1.4 | 91 ± 1 | 44 | 3.8 |
| *S_Zn_FeOOH_* | 136 ± 2.8 | 85 ± 1 | 41 | 8.7 |

The addition of PVP led to thicker membranes with higher porosity, likely due to increasing the viscosity of the casting solution (cf. Table 1 in the main text). Crucially, PVP did not affect particle integration, as the Fe loading in the *S_FeOOH* membrane was comparable to the PVP-free *M_Fe* membrane (cf. Table 1). Although the Fe loadings were consistent across all samples, the incorporation efficiency of the secondary metals varied. This variability may be caused by interactions between iron ions and secondary metals or galvanic displacement reactions during the *in situ* reduction. Despite these variations in doping levels, bimetallic catalysts were successfully synthesized in all cases.


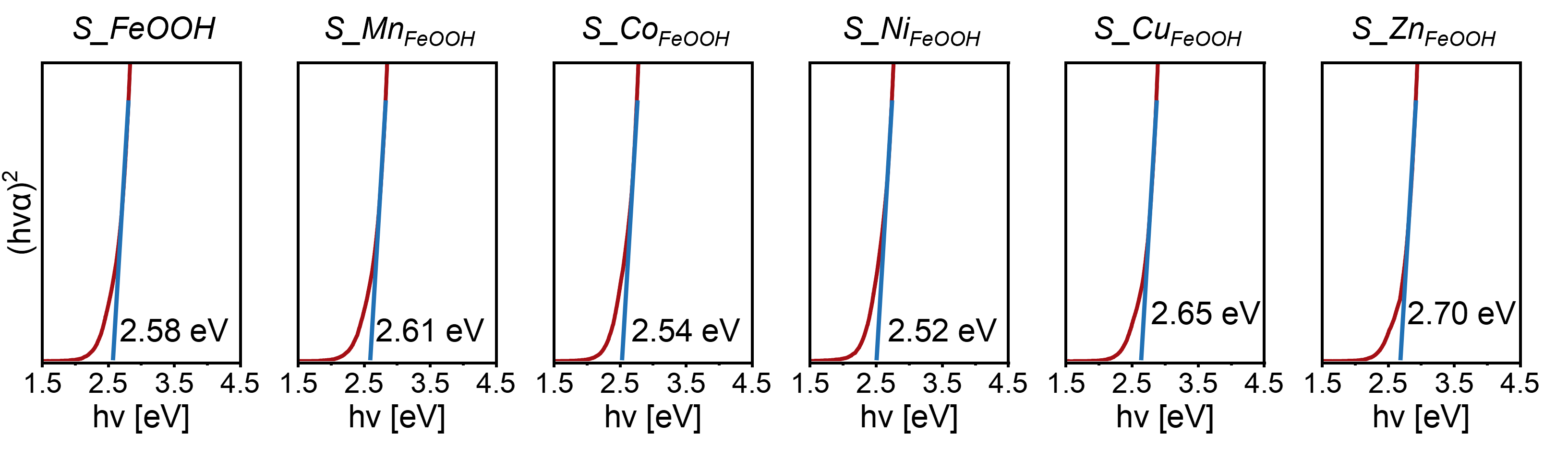
To characterize the membrane-immobilized metal-doped FeOOH species we determined their band gap energy (Figure S12).

*Figure S12. Optical band gap energy of the metal-doped FeOOH species. Tauc plots were generated from UV-Vis absorption spectra of the membrane-immobilized particles after dissolving the membrane matrix in NMP.*

The band gap of *S_FeOOH* was not drastically altered by doping, suggesting that FeOOH remained the primary oxide phase in all catalysts. Notably, the color of the membranes after the post-treatment oxidation was also not significantly affected by the introduction of the secondary metals. However, subtle but distinct shifts in the band gap energy were observed for each secondary metal dopant. These shifts suggest that the secondary metals were successfully integrated into the FeOOH bulk lattice. Notably, this also demonstrates the versatility of the *all-in-one* method and indicates its potential for preparing alloy or mixed metal catalysts.

For the validation of our framework, we further altered both the reaction conditions and the quantification method for determining catalytic activity. We immersed two 18 mm samples of each membrane separately in DI water with 25 mg/L diclofenac and 5 mM PS, followed by shaking at 30°C for 20 h. Additionally, we conducted the same reaction under the same conditions, except in an ionic water matrix (pH ~8, Table S8).

*Table S8: Composition of the ionic water matrix.*

| Salt | Concentration [mg/L] |
| --- | --- |
| NaHCO_3_ | 50 |
| Na_2_HPO_4_ | 47 |
| KH_2_PO_4_ | 47 |
| MgCl_2_ | 32 |
| NH_4_Cl | 26 |
| CaCl_2_ | 10 |

After the 20 h reaction, we determined the amount of sulfate released into the reaction solution using ion chromatography (Metrohm 883 Basic IC plus). During activation of PS by the catalysts, each PS molecule should be cleaved into a sulfate radical and a sulfate anion. Therefore, the amount of generated sulfate anions should directly correlate with the amount of activated PS.

Importantly, for this validation, we first used the framework to predict that M(II)-doped FeOOH species would exhibit catalytic activity for oxidant activation in the following order: Mn(II)_FeOOH_ > Ni(II)_FeOOH_ > Zn(II)_FeOOH_ > Co(II)_FeOOH_ > Cu(II)_FeOOH_ > Fe(III)OOH. Only after this prediction, we designed, synthesized, and experimentally evaluated the catalysts. However, it is important to note that this evaluation assumes that all secondary metals were incorporated as M(II)-OH species into the FeOOH bulk and that the surface properties are similar across the different catalysts. Therefore, this validation provides only initial supporting evidence for the validity of the framework. For conclusive validation, further, fully characterized catalysts will need to be evaluated.

We assumed that the coordination of M(II)-OH sites mirrors that of Fe(III)-O sites (CN=6 in the bulk, CN=5 at the surface). Based on this, we first calculated the base IE (Equation S4) of the secondary metal sites using the band gap of the respective bulk oxide (cf. Figure S12; this base IE is lower for Co(II) than for Zn(II) due to the higher band gap energy of *S_Zn_FeOOH_*) and their Shannon ionic radii (Equation S3). A comparison of this base IE with the experimentally determined sulfate concentration in solution after the reaction already shows a strong qualitative agreement with the activity prediction (Figures S13a and S14a), even before accounting for differences in secondary metal loading and without applying our modeling procedure.

To further quantify the catalytic activity, we calculated the TOF of the secondary metals for sulfate formation from PS (*TOF_Sulfate_*; mg released sulfate per mg secondary metal per h; cf. Table S7) and used it to model the *IE_MAS_* values of each M(II) site as described in Section S8. For the reaction in DI water (Figure S13b), this produced *IE_MAS_* values for all secondary metals that closely match their observed *TOF_Sulfate_* values. Even for the reaction in the ionic water matrix (Figure S14b), we obtained a relatively good match between *IE_MAS_* and *TOF_Sulfate_* values, with only minor variations between modeling and experimental data.

*Figure S13: a) Sulfate formation from PS (5 mM) after 20 h reaction in a solution (10 mL) of DF (25 mg/L) in DI water using the membrane-immobilized doped FeOOH species as immersed catalysts (18 mm). The unfitted base IE (cf. Equation S4) of the M(II) species was*
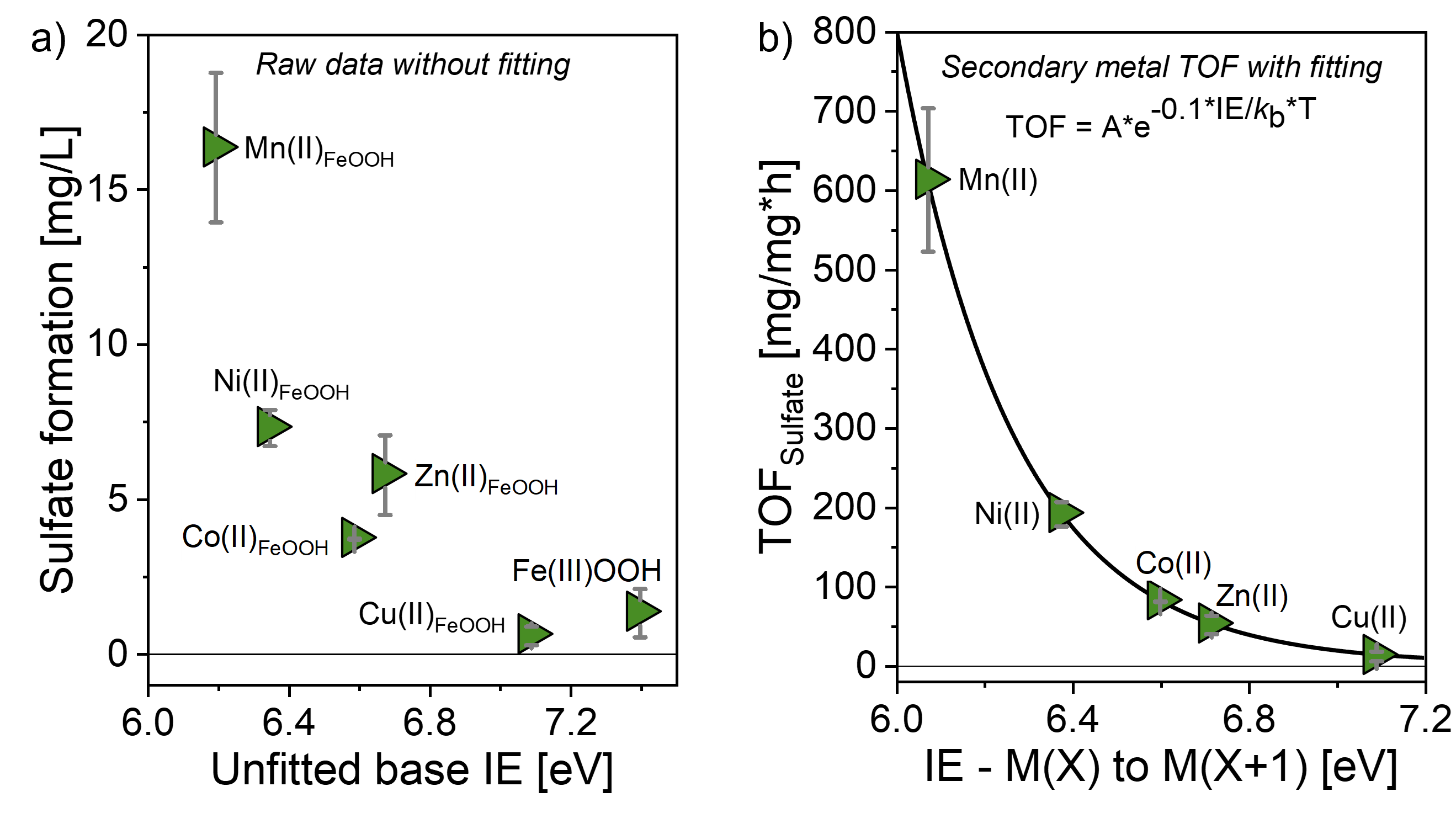
*calculated from their Mulliken electronegativity (cf. Equation S3) and the band gap energy of the respective doped bulk oxides (cf. Figure S12). b) Modeled IE_MAS_ of the secondary M(II) sites in the membrane-immobilized doped FeOOH catalysts using the TOF_Sulfate_ values as input. Each reaction was conducted with two samples of each membrane and the average and standard deviations are shown.*

*Figure S14: a) Sulfate formation from PS (5 mM) after 20 h reaction in a solution (10 mL) of DF (25 mg/L) in the ionic water matrix (cf. Table S8) using the membrane-immobilized doped*
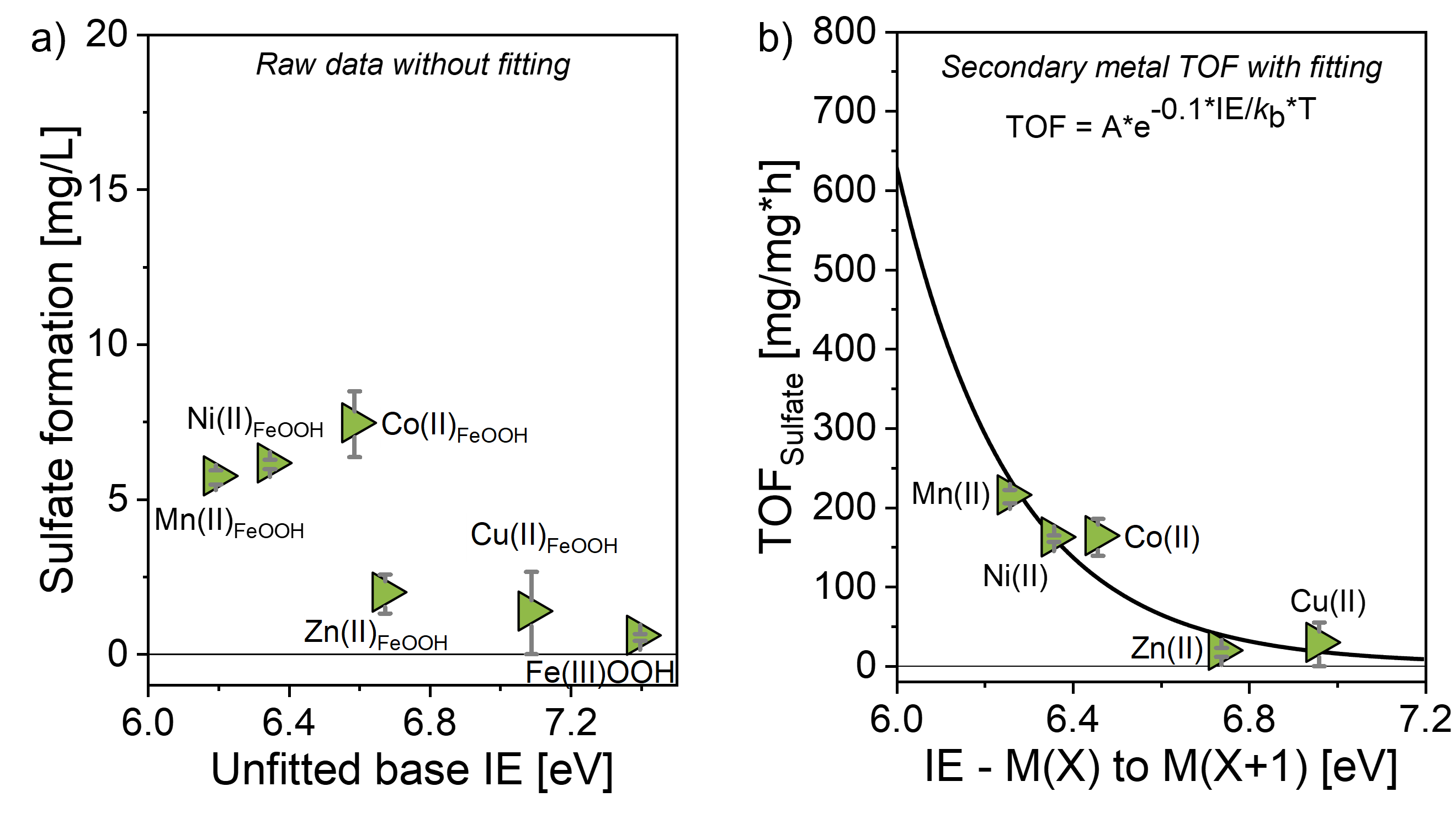
*FeOOH species as immersed catalysts (18 mm). The unfitted base IE (cf. Equation S4) of the M(II) species was calculated from their Mulliken electronegativity (cf. Equation S3) and the band gap energy of the respective doped bulk oxides (cf. Figure S12). b) Modeled IE_MAS_ of the secondary M(II) sites in the membrane-immobilized doped FeOOH catalysts using the TOF_Sulfate_ values as input. Each reaction was conducted with two samples of each membrane and the average and standard deviations are shown.*

**References**

[1] S.G. Bratsch, Standard electrode potentials and temperature coefficients in water at 298.15 K, J. Phys. Chem. Ref. Data 18 (1989) 1–21.

[2] T. Misawa, K. Hashimoto, S. Shimodaira, The mechanism of formation of iron oxide and oxyhydroxides in aqueous solutions at room temperature, Corros. Sci. 14 (1974) 131–149. https://doi.org/10.1016/S0010-938X(74)80051-X.

[3] S.A.H. Hesaraki, M. Ulbricht, L. Fischer, *All-in-one* fabrication of NiO nanorods/carbon-containing porous catalytic polymer membranes for persulfate-facilitated oxidation-adsorption of diclofenac in flow–through, Chem. Eng. J. 498 (2024) 155266. https://doi.org/10.1016/j.cej.2024.155266.

[4] S.F. Matar, G. Campet, M.A. Subramanian, Electronic properties of oxides: Chemical and theoretical approaches, Prog. Solid State Chem. 39 (2011) 70–95. https://doi.org/10.1016/j.progsolidstchem.2011.04.002.

[5] X. Zhang, T. Liu, L. Zhu, J. Guan, Y. Lu, T.W. Keal, J. Buckeridge, C.R.A. Catlow, A.A. Sokol, Bulk and Surface Contributions to Ionisation Potentials of Metal Oxides, Angew. Chem. Int. Ed. 62 (2023) e202308411. https://doi.org/10.1002/anie.202308411.

[6] R.D. Shannon, C.T. Prewitt, Effective ionic radii in oxides and fluorides, Acta Crystallogr. B 25 (1969) 925–946. https://doi.org/10.1107/S0567740869003220.

[7] S. Lindskog, Structure and mechanism of carbonic anhydrase, Pharmacol. Ther. 74 (1997) 1–20. https://doi.org/10.1016/S0163-7258(96)00198-2.

[8] J. Leichtweis, E. Carissimi, U. Hagemann, M. Ulbricht, L. Fischer, NiFe2O4/biochar decorated porous polymer membranes for the flow-through photo-Fenton degradation of tetracycline, Chem. Eng. J. (2023) 147203. https://doi.org/10.1016/j.cej.2023.147203.

[9] J. Leichtweis, Y. Vieira, E. Carissimi, M. Ulbricht, L. Fischer, Photocatalytic and persulfate-facilitated tetracycline degradation using NiFe2O4/carbon/polymer membranes: Activity and stability during flow-through, Appl. Surf. Sci. 669 (2024) 160603. https://doi.org/10.1016/j.apsusc.2024.160603.

[10] O. Muller, R. Wilson, W. Krakow, δ-FeO(OH) and its solid solutions, J. Mater. Sci. 14 (1979) 2929–2936. https://doi.org/10.1007/BF00611477.
